# Remapping of the belted phenotype in cattle on BTA3 identifies a multiplication event as the candidate causal mutation

**DOI:** 10.1186/s12711-018-0407-9

**Published:** 2018-07-06

**Authors:** Sophie Rothammer, Elisabeth Kunz, Stefan Krebs, Fanny Bitzer, Andreas Hauser, Natalia Zinovieva, Nikolai Klymiuk, Ivica Medugorac

**Affiliations:** 1 Population Genomics Group, Department of Veterinary Sciences, LMU Munich, Veterinärstr. 13, 80539 Munich, Germany; 2 Laboratory for Functional Genome Analysis, Gene Center Munich, LMU Munich, Feodor-Lynen-Str. 25, 81377 Munich, Germany; 3 The L.K. Ernst Institute of Animal Husbandry, Moscow Region, Russian Federation; 4 Chair for Molecular Animal Breeding and Biotechnology, LMU Munich, Hackerstr. 27, 85764 Oberschleissheim, Munich, Germany

## Abstract

**Background:**

It has been known for almost a century that the belted phenotype in cattle follows a pattern of dominant inheritance. In 2009, the approximate position of the *belt* locus in Brown Swiss cattle was mapped to a 922-kb interval on bovine chromosome 3 and, subsequently, assigned to a 336-kb haplotype block based on an animal set that included, Brown Swiss, Dutch Belted (Lakenvelder) and Belted Galloway individuals. A possible candidate gene in this region i.e. *HES6* was investigated but the causal mutation remains unknown. Thus, to elucidate the causal mutation of this prominent coat color phenotype, we decided to remap the belted phenotype in an independent animal set of several European bovine breeds, i.e. Gurtenvieh (belted Brown Swiss), Dutch Belted and Belted Galloway and to systematically scan the candidate region. We also checked the presence of the detected causal mutation in the genome of belted individuals from a Siberian cattle breed.

**Results:**

A combined linkage disequilibrium and linkage analysis based on 110 belted and non-belted animals identified a candidate interval of 2.5 Mb. Manual inspection of the haplotypes in this region identified four candidate haplotypes that consisted of five to eight consecutive SNPs. One of these haplotypes overlapped with the initial 922-kb interval, whereas two were positioned proximal and one was positioned distal to this region. Next-generation sequencing of one heterozygous and two homozygous belted animals identified only one private belted candidate allele, i.e. a multiplication event that is located between 118,608,000 and 118,614,000 bp. Targeted locus amplification and quantitative real-time PCR confirmed an increase in copy number of this region in the genomes of both European (Belted Galloway, Dutch Belted and Gurtenvieh) and Siberian (Yakutian cattle) breeds. Finally, using nanopore sequencing, the exact breakpoints were determined at 118,608,362 and 118,614,132 bp. The closest gene to the candidate causal mutation (16 kb distal) is *TWIST2*.

**Conclusions:**

Based on our findings and those of a previously published study that identified the same multiplication event, a quadruplication on bovine chromosome 3 between positions 118,608,362 and 118,614,132 bp is the most likely candidate causal mutation for the belted phenotype in cattle.

**Electronic supplementary material:**

The online version of this article (10.1186/s12711-018-0407-9) contains supplementary material, which is available to authorized users.

## Background

Breeders as well as geneticists have been interested in the inheritance of obvious coat color patterns for many decades [1–3]. Besides plain-colored and spotted phenotypes that more or less uniformly affect the whole body, there are also “segmental body traits” that seem to affect only a distinct part of the animal. Examples for such segmental coat color phenotypes are the white head in Simmental or Hereford cattle, the half-black, half-white coat color of the Valais Blackneck goat, and the belt pattern, which exists in various species.

In general, the belt pattern can be described as a white band of varying width around the midsection of the body [4] but does not always encircle the body completely, and is considered to result from a lack of melanocytes [5]. Olson calls it “one of the most striking white-spotting mutants” and believes that modifying genes are responsible for the width of the belt [4]. The belt pattern is well described in mice, pigs and cattle and its genetic determinism has been extensively studied. In the mouse, Rao et al. [6] reported a recessive belt pattern that is caused by mutations in the *ADAMTS20* gene. The belt in Hampshire swine was first studied in 1907 by Spillman [3] who hypothesized that it was due to the complementary action of at least two factors. Many other studies followed and considered that this trait displayed a dominant mode of inheritance [1, 7, 8]. In 1999, Giuffra et al. [9] confirmed the dominant inheritance of the belt pattern in Hampshire swine and identified the *belt* locus as the fourth allele at the *KIT* locus on pig chromosome 8. To date, it is not clear if this *KIT* allele also causes the belt phenotype in Chinese pig breeds [10].

For belted cattle, a dominant inheritance of this trait was reported as early as 1921 [11]. The monogenic dominant mode of inheritance was confirmed in 2001 by Schmutz et al. [12]. However, the physical appearance of a belt is apparently not inherited in a simple way because animals that are homozygous at the *belt* locus do not necessarily have a more perfect belt than heterozygous animals [5, 12].

In 2009, the *belt* mutation in Gurtenvieh (GUV) cattle (which is the name of belted Brown Swiss cattle) was mapped to the telomeric region of *Bos taurus* chromosome 3 (BTA3). Since the former candidate genes, *KIT* and *ADAMTS20*, are positioned on other chromosomes than BTA3, they were excluded as causative genes [5]. After remapping of the mutation and haplotype analysis, these authors reported that it was most likely located in a 922-kb segment on BTA3. In a second study, two additional belted breeds were analyzed, i.e. Belted Galloway (BGA) and Lakenvelder (Dutch Belted, DBE). According to the histories of these two breeds, their belt phenotype could originate from Gurtenvieh cattle [13, 14]. For each breed analyzed, a single belt-associated haplotype was identified. A comparison of these haplotypes revealed four short haplotype blocks of which the most extended block (336 kb) spanned nine SNPs and contained one potential candidate gene that encodes the developmental transcription factor HES6. Although the *HES6* coding sequence was completely re-sequenced, no belt-associated polymorphism was detected [13]. Thus, when we decided to remap the *belt* locus in order to localize the underlying mutation, the causal polymorphism for the belted phenotype in cattle was still unknown. However, during the course of our analyses, which led to the identification of a strong candidate causal mutation, Awasthi Mishra et al. published an article [15] that describes the same mutation as the most likely cause for the belt pattern. Thus, our study confirms the findings of Awasthi Mishra et al. [15] by using an independent animal set that includes a Siberian breed in addition to European cattle breeds and strengthens the assumption that the approximately 6-kb copy number variation (CNV) upstream of the *TWIST2* gene is indeed the causative mutation of the belted phenotype in cattle. Moreover, we provide detailed information on the repetitive structure of this special locus.

## Methods

### Animal samples

**Fig. 1:**
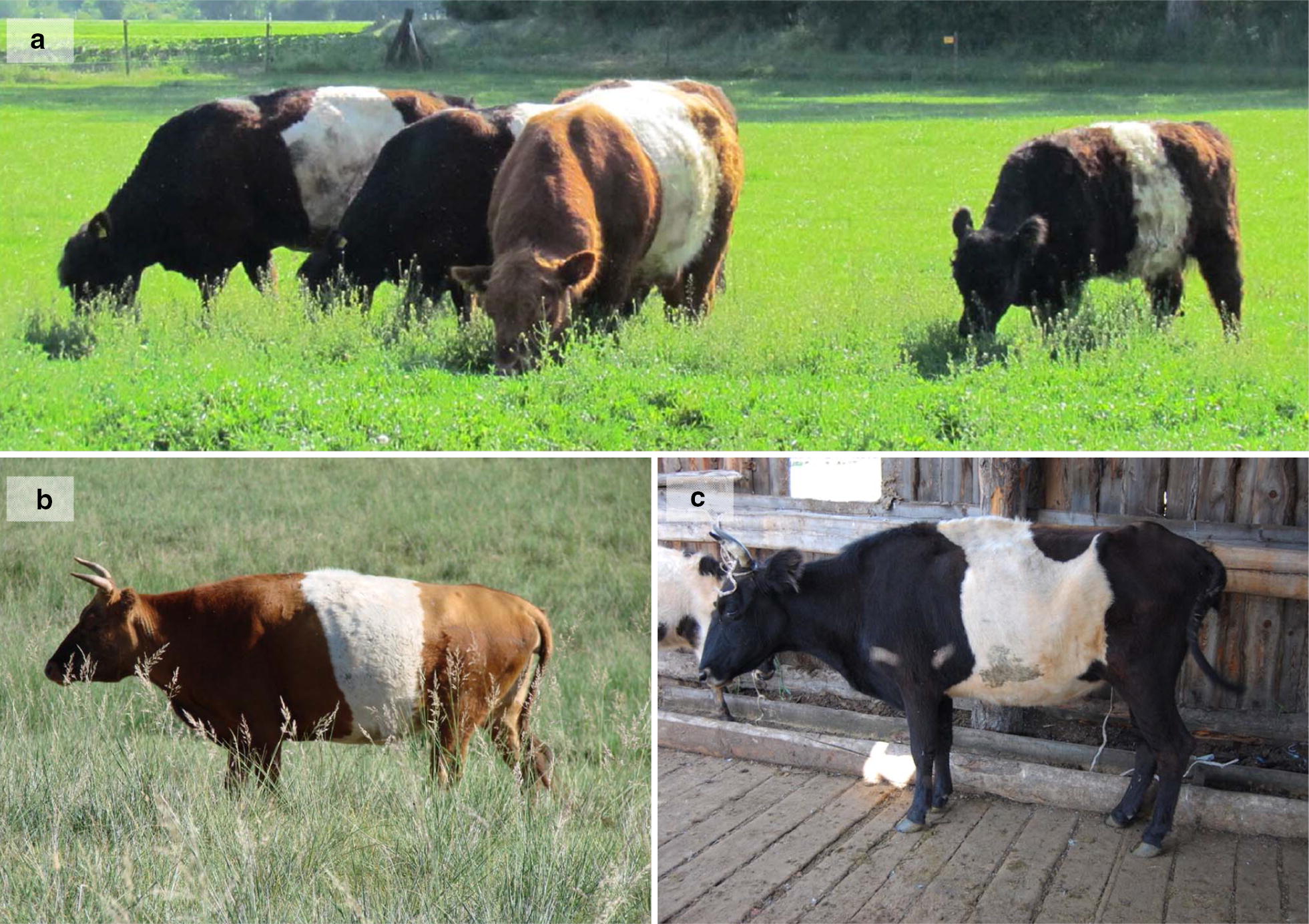
Belted cattle. **a** Red Belted Galloway bull with three Black Belted Galloway cows, for which the varying size of the belt is obvious. **b** Belted Mongolian cattle. **c** Belted Yakutian cattle

For this study, 121 animals were analyzed, of which 117 were sampled from the following breeds, i.e. 43 Belted Galloway (Fig. 1), 29 Black or Red Galloway GAL), 26 Dutch Belted, and 19 Gurtenvieh, and four were analyzed from two non-belted offspring of Gurtenvieh parents and two belted and non-belted full siblings descending from a Gurtenvieh (dam) × Pinzgauer cattle (sire) cross. However, five Gurtenvieh and six Belted Galloway animals were excluded from the animal set used for remapping because of an “atypical” phenotype. Thus, the final animal set comprised 110 animals of which 78 were belted and 32 were plain-colored.

When available, we used pedigree data to differentiate between homozygous and heterozygous belted animals. Homozygosity was assumed as likely if all the ancestors in the last three generations were belted. Heterozygosity was inferred when the animals themselves originated from crosses between belted and black Galloway or between belted and non-belted breeds, or if they had at least one non-belted offspring. Thus, it was possible to assign the 110 animals to four groups: (i) non-belted, (ii) heterozygous belted, (iii) likely homozygous belted, and (iv) with a belted phenotype but a completely ambiguous genotype. We assumed that the Dutch Belted animals were homozygous at the *belt* locus. These group affiliations were taken in account in the variance component method that combines linkage disequilibrium (LD) and linkage analysis (cLDLA) (see section “Remapping of the belted phenotype by *cLDLA* procedure”). Since the belt pattern in cattle is a dominant phenotype, only the heterozygous classification can be assumed to be accurate, whereas the homozygous classification can be incorrect in some cases, which is why we called this group “likely homozygous”. However, our previous analyses on the mapping of the Weaver disease [16], showed that such misclassifications do not hinder effective mapping by the procedure applied here.

For qPCR analysis, samples of eight belted and two non-belted Russian Yakutian cattle (RUY), which belong to the Mongolian-Turano group of taurine cattle, were also collected. Interestingly, the belt phenotype of most Yakutian cattle was less accurate than those of European breeds (Fig. 1c). However, since belted Turano cattle with a European-like phenotype are present in Mongolia (Fig. 1b; J. Peters personal communication), the phenotypic difference between the Yakutian cattle and European breeds might not be due to a distinct belted mutation in the North-East Asian Turano cattle but rather to various coat color phenotypes segregating in Yakutian cattle.

### Genotyping data, quality control and haplotype reconstruction

DNA was extracted from blood, hair root samples or semen, and genotyped using either version 1 or version 2 of the BovineSNP50 Genotyping BeadChip (Illumina Inc., San Diego, USA). Physical positions of all SNPs were based on the reference assembly of the bovine genome UMD3.1 [17, 18]. The following SNPs were excluded from further analysis: (i) SNPs with a call rate lower than 95%, (ii) SNPs that displayed frequent paternity conflicts in animals with known paternity (i.e. SNPs showing Mendelian error rates above ~ 0.2%), (iii) SNPs with an unknown position according to the reference assembly UMD3.1, (iv) SNPs with a minor allele frequency (MAF) lower than 0.025, and (v) SNPs located on bovine chromosomes other than BTA3 since the *belt* locus is already mapped to BTA3 [5, 13]. After this filtering process, 2111 SNPs remained for further analyses.

After quality control, haplotypes were reconstructed and missing genotypes were imputed using the Hidden Markov model implemented in BEAGLE 3.3.2 [19]. To improve haplotype reconstruction and imputation of missing alleles, SNP genotypes of more than 9000 animals from previous studies were included, although these animals had no direct relevance to our study.

### Estimation of unified additive relationships and locus IBD

Within the mixed linear model used to remap the belted phenotype, locus IBD (LocIBD) was used as a correction for local haplotype relationships, while the unified additive relationships (UAR) were used to correct for population stratification and familial relationships. Hence, UAR were estimated between all animals [20], then all the principal components of the UAR matrix were determined using R [21], and the number of principal components that explained more than 95% of the genetic variance was ascertained using the R package paran [22]. Thus, genome-wide relationships were accounted for by using the 60 most significant principal components of the UAR matrix.

For local haplotype relationships, sliding windows of 40 consecutive SNPs along BTA3 were used. At each window midpoint (i.e. between SNP 20 and 21), the LocIBD was estimated [23] and, as shown in Lee and Van der Werf [24], the resulting haplotype-based IBD matrices were converted into diplotype relationship matrices (called ${\mathbf{D}}_{{{\mathbf{RM}}}}$ hereafter).

### Remapping of the belted phenotype by the cLDLA procedure

Estimation of ${\mathbf{D}}_{{{\mathbf{RM}}}}$ for sliding windows and remapping were carried out by a procedure that is equivalent to the combined linkage/linkage disequilibrium mapping method reported by Meuwissen et al. [25] and has already been applied for QTL mapping in swine [26], fine-mapping of the Weaver disease in Braunvieh [16], and for mapping fertility traits in Holstein cattle [27].

Finally, ASReml [28] was used for a variance component analysis at the midpoint of each sliding window. The mixed linear model included random phenotype effects that were based on ${\mathbf{D}}_{{{\mathbf{RM}}}}$ and the 60 principal components used as covariates to account for polygenic effects. To prepare the vector of phenotypes, all plain-colored animals were assigned the numerical value “1”, heterozygous belted animals “2”, and likely homozygous belted animals “3”. Belted animals with a completely ambiguous genotype status were set between heterozygous and likely homozygous belted and thus were assigned “2.5”. The resulting model was:

$$ {\mathbf{y}} = {\mathbf{X}}{\varvec{\upbeta}} + {\mathbf{Zq}} + {\mathbf{e}}, $$

where **y** is the vector of phenotypes, ${\varvec{\upbeta}}$ is a vector of fixed effects (including the overall mean $\mu$ and the 60 principal components), **q** is a vector of random additive genetic effects due to the belt locus with ${\mathbf{q}}\sim\,N\left( {0, {\mathbf{D}}_{{{\mathbf{RM}}p}}\upsigma_{\text{q}}^{2} } \right)$, where ${\mathbf{D}}_{{{\mathbf{RM}}p}}$ is the diplotype relationship matrix at position *p* of the putative *belt* locus, and **e** a vector of random residual effects with ${\mathbf{e}}\sim\,N\left( {0, {\mathbf{I}}\upsigma_{\text{e}}^{2} } \right)$, where **I** is an identity matrix.

The random effects **q** and **e** were assumed to be uncorrelated and normally distributed and their variances ($\upsigma_{\text{q}}^{2} ,$ and $\upsigma_{\text{e}}^{2}$) were simultaneously estimated using ASReml [28].

The likelihood ratio test statistic (${\text{LRT}} = - 2(\log \left( {L_{0} } \right) - { \log }\left( {L_{P} } \right)$), which is known to be X^2^-distributed with one degree of freedom [29], was calculated from the logarithm of the likelihood that was estimated for both the model with $\left( {{ \log }\left( {L_{P} } \right)} \right)$ and without QTL effect ($\log \left( {L_{0} } \right)$; corresponding to the null hypothesis).

Finally, the chromosome-wide highest LRT peak (LRT_max_) was identified, and the respective confidence interval of the *belt* locus was determined using the 2-LOD (log off odds; one LOD = 4.605 LRT) drop-off criterion [30, 31]. Since each value represents the position at the midpoint of a 40-SNP sliding window, the initial confidence interval was extended by 20 SNPs in each direction. For this extended confidence interval, positional candidate genes were identified using the Ensembl release 86 [32]. To identify the most likely position of the *belt* locus, the extended confidence interval was searched manually across the whole animal set for haplotypes that were at least heterozygous in belted and absent in plain-colored animals. Haplotypes within the extended confidence interval that met these criteria are called inner candidate haplotypes (*IC*-*Hap*) in the following sections.

### Next-generation sequencing

DNA samples from a likely homozygous Belted Galloway individual, a Dutch Belted individual and a heterozygous belted cross individual between Gurtenvieh and Pinzgauer were sequenced at the Laboratory for Functional Genome Analysis of the LMU Munich. Libraries were prepared from genomic DNA after ultrasonic fragmentation (Covaris M220 (75 s, 20% duty factor)) using the Accel-DNA 1S kit (Swift Biosciences, Ann Arbor, USA). Libraries were sequenced in 100-bp paired-end mode on a HiSeq 1500 (Illumina, San Diego, USA). All reads were aligned to the reference sequence (UMD 3.1) using the Burrows-Wheeler Aligner (BWA) [33]. Variant calling and filtering for variants that were homozygous and different from the reference sequence in the Belted Galloway and Dutch Belted animals and, at the same time, heterozygous in the belted cross individual, were performed with SAMtools mpileup [34] and VarScan [35] for the whole extended confidence interval.

To reduce the list of candidate causal variants, we used four Holstein–Friesian bulls that had previously been sequenced for another project and five animals for each of the Simmental, Jersey and Angus breeds that had been sequenced within the 1000 Bull Genomes Project [36]. Thus, we could exclude candidate causal variants that were heterozygous or homozygous in at least one of these animals.

Moreover, sequence data that matched an *IC*-*Hap* were visually inspected using the Integrative Genomics Viewer (IGV) [37, 38] to check for possible rearrangements (e.g. extended InDel) that cannot be detected by the applied automated variant calling and filtering process (mpileup [34] and VarScan [35]).

### Resequencing of BAC clones

To ensure that the reference sequence of the candidate segment (see section “Results of NGS”) that was identified by NGS was correctly assembled, two BAC clones (CH240-library: CH240-363B2, CH240-104M22) that mapped to the region of interest according to the NCBI CloneFinder [39] were re-sequenced. Libraries were prepared for both Illumina paired-end sequencing and nanopore 2D sequencing. Reads from nanopore sequencing (MinION Mk1B, R9 flowcell, Oxford Nanopore Technologies, Oxford, Great Britain) were assembled with the CANU assembler [40] and error correction was performed with Illumina 100-bp paired-end reads of the same clone using BWA [33], mpileup (SAMtools package) [34], VarScan [35], and a custom script for editing the variants called by VarScan in the reference FASTA file.

### Quantitative real-time PCR

To determine the relative copy number ratio of the candidate segment between belted and non-belted animals, three qPCR primer pairs within the candidate segment (Fig. 4 and Table 1) and two qPCR primer pairs flanking the candidate segment (belt_b1, belt_d2) were designed. In addition, two qPCR primer pairs were designed for reference sites within the *LPO* and *PRPN* genes. All seven qPCR were run under optimized conditions (Table 2) on the LightCycler^®^ 96 System (Roche, Rotkreuz, CH) using FastStart Essential DNA Green Master (Roche, Rotkreuz, CH). The copy numbers of the investigated segment were determined from a standard curve derived from serial dilutions of a reference DNA. The relative copy number ratio (candidate segment to the reference site) was calculated for all constellations.

**Table 1:** Primers used for qPCR

ID	Sequence	Region
belt1_f	CCGTGGACAAGAGGAAAATA	Candidate segment
belt1_r	GGCTGACTGCGTTTTTAGTG	
belt2_f	TGCCAGAGGATGAGTGTGAG	Candidate segment
belt2_r	CAGACCCAGGAGCCATTAAG	
belt3_f	TAGATGCTTCTGTTGACCAC	Candidate segment
belt3_r	ATGTCTCACCGCCACTGTC	
belt_b1_f	GTGGGAATGGCGGTCTAAAT	Flanking candidate segment
belt_b1_r	CTGACCTTGTTCCCTCTTCAC	
belt_d2_f	GCTCAGCATCCCTGGTGATT	Flanking candidate segment
belt_d2_r	ACTGGACTGCCAGGGAATTG	
lpo_f	ATGCCTTCCAGGCCAACAAC	Reference
lpo_r	GAGCTCTACTGCACAGTGTG	
prpn_f	GATGCCACTGCTATGCAGTC	Reference
prpn_r	CACGTCACTCCACATGGCCACA

**Table 2:** qPCR conditions

	prpn	lpo1	belt1	belt2	belt3	belt_b1	belt_d2
Annealing temperature (°C)	61	63	63	61	63	63	65
SYBR green	6.25	6.25	6.25	6.25	6.25	6.25	6.25
UNG	0.075	0.075	0.075	0.075	0.075	0.075	0.075
Forward (5 µM)	0.5	0.3	0.5	0.3	0.3	0.2	0.2
Reverse (5 µM)	0.5	0.5	0.5	0.5	0.5	0.3	0.2
Template	2.5	2.5	2.5	2.5	2.5	2.5	2.5
H_2_O	2.675	2.875	2.675	2.875	2.875	3.175	3.275

### Targeted locus amplification

Targeted locus amplification (TLA) is a method for targeted re-sequencing that is based on crosslinking of physically proximal sequences. Thus, this technique can generate DNA libraries that cover more than 100 kb of contiguous DNA on either side of a primer pair [41, 42]. An advantage of the TLA method is that it does not depend on detailed prior locus information. Thus, it is particularly useful when there is doubt about the overall correctness of the reference genome sequence in the target region and/or when extended rearrangements are assumed to have occurred within the target region [42] as was assumed for the candidate segment in belted animals. For re-sequencing with TLA, leukocytes were isolated from fresh EDTA-blood samples of a Belted and a Black Galloway individual according to the protocol provided by Cergentis (NL) [41]. Locus amplification, sequencing and mapping were performed by Cergentis.

### Long-read whole-genome sequencing

The Dutch Belted animal, LKF08, was chosen for whole-genome nanopore sequencing in order to further elucidate the structure of the expanded repeat region. Five nanograms of needle-sheared DNA (obtained by passing the sample through a 20 gauge needle five times) were used for library preparation with the Oxford Nanopore Kit for 1D libraries (LSK108). The DNA was end-repaired with NebNext UltraII End repair module and simultaneously damage-repaired with the FFPE repair kit (both from New England Biolabs). The reaction was carried out with 24 μL of DNA, 1.75 μL of end repair buffer, 1.75 μL of FFPE buffer, 1.5 μL of end repair enzyme mix and 1 μL of FFPE repair enzyme mix. This reaction mix was incubated for 5 min at 20 °C and 5 min at 65 °C. Then, 20 μL of AMX adapter and 30 μL of NebNext UltraII Ligation mix were added and incubated for 10 min at room temperature. The ligated library was purified with Ampure XP beads and prepared for sequencing following the instructions of the Oxford Nanopore kit for 1D libraries. The final sequencing mix was loaded onto a PromethION flow cell and sequenced for 60 h on a PromethION alpha version instrument (ONT, Oxford UK). The resulting data were base-called with Albacore 2.1.7. The resulting 6.75 Gbp of fastq data were mapped to UMD3.1.1 with minimap2 (Li H (2017) Minimap2: fast pairwise alignment for long nucleotide sequences. arXIV: 1708.01492). Reads that mapped to the belted candidate region were extracted and assembled with CANU [40].

## Results

### Results of *cLDLA* remapping and manual identification of candidate haplotype(s)

**Fig. 2:**
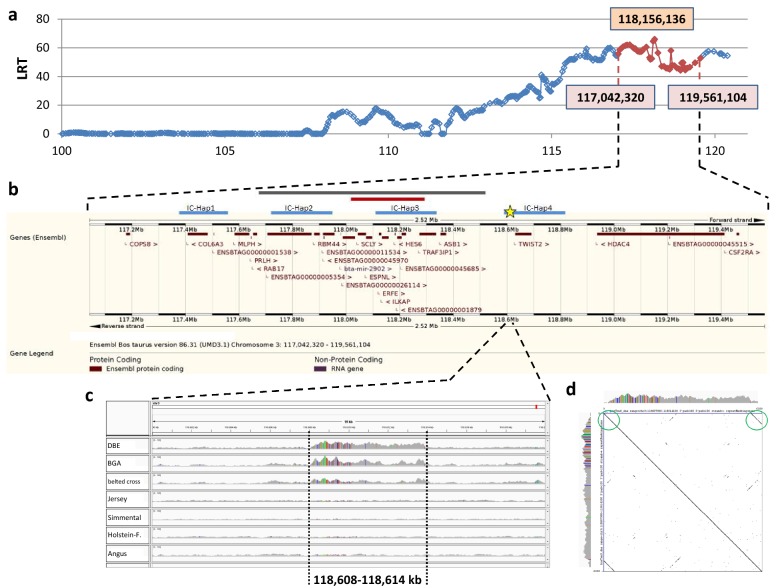
*cLDLA* and NGS results. **a** Results of the *cLDLA*. The extended confidence interval (eCI) is shown in red; positions of the borders of the eCI and the genome-wide maximal LRT value are provided. **b** Ensembl genome browser result for the eCI. The grey bar above indicates the critical interval identified by Drögemüller et al. [5], the red bar indicates the candidate interval according to Drögemüller et al. [13], the blue bars indicate the inner candidate haplotypes (i.e. *IC*-*Hap*1 to 4 from left to right) identified by manual haplotype analysis of the eCI; the position of the 6-kb candidate segment (*Belt*_*Multi6kb*_) is marked with a yellow star; as shown, *Belt*_*Multi6kb*_ is located next to the previously reported candidate haplotypes. **c** NGS results of *Belt*_*Multi6kb*_ and surrounding regions. The first two lines show the coverage of homozygous belted animals (1. Dutch Belted = DBE, 2. Belted Galloway = BGA) and the third line shows a heterozygous belted crossbred, while the other lines show non-belted control animals (4. Jersey, 5. Simmental, 6. Holstein–Friesian, 7. Angus). SNPs are indicated by colored vertical lines within the coverage. The approximate extension of the multiplication event is provided. **d** Dotplot of the reference sequence of the 6-kb candidate segment against itself. Green circles highlight the obvious sequence similarity at the end and beginning of *Belt*_*Multi6kb*_

As shown in Fig. 2a, the LRT_max_ position was detected at 118,156,136 bp and the extended confidence interval ranged from 117,042,320 to 119,561,104 bp on BTA3. Figure 2b gives an overview of the positional candidate genes according to the Ensembl genome browser and also indicates the candidate causal region identified by Drögemüller et al. [13].

To narrow down the true position of the *belt* locus, the haplotypes of the extended confidence interval, which was defined by the cLDLA analysis, were visually analyzed. Therefore, haplotypes consisting of 60 SNPs that covered the whole extended confidence interval were sorted by breed and by assumed belt genotype (see Additional file 1). First, two candidate haplotypes that perfectly fitted the phenotypes of Belted Galloway and a third haplotype that matched the situation in Dutch Belted cattle were identified. Then, two other common haplotypes were found in Gurtenvieh cattle. Assuming that the belted phenotype was introgressed into Dutch Belted and Belted Galloway by Gurtenvieh individuals, we hypothesized that the *belt* locus resides in a region common to these five haplotypes. These five haplotypes overlapped with four inner candidate haplotypes (*IC*-*Hap1*-*4*) that extended across five to eight SNPs (Table 3). Among these, *IC*-*Hap3* overlapped with the candidate haplotype identified by Drögemüller et al. [13], *IC*-*Hap1* and *IC*-*Hap2* were located proximal and *IC*-*Hap4* was located distal to this region (Fig. 2b).

**Table 3:** Inner candidate haplotypes detected by manual inspection of the extended confidence interval

Haplotype	Number of SNPs	Start (bp)	End (bp)
IC-Hap1	6	117,378,957	117,557,450
IC-Hap2	8	117,725,426	117,960,375
IC-Hap3	6	118,145,216	118,346,051
IC-Hap4	5	118,592,298	118,813,014

### Results of next-generation sequencing

#### Automated variant calling and filtering

Filtering of all the variants included in the combined confidence interval region to select only those that were homozygous in both the Belted Galloway and Dutch Belted animals and heterozygous in the Gurtenvieh crossbred animal produced a list of 298 candidate variants (15 InDel and 283 SNPs). However, a comparison with control animals showed that, for each of these polymorphisms, at least one animal in the non-belted control group was also heterozygous or homozygous for the respective variant. Consequently, all these variants were excluded as candidate causal mutations.

#### Visual inspection of IC-Haps

Visual inspection identified a ~ 6-kb segment with an elevated read coverage (called *Belt*_*Multi6kb*_ hereafter) that was not observed in any of the non-belted animals (see Fig. 2c). This segment extends from ~ 118,608,000 to 118,614,000 bp on BTA3 (in both reference genomes bosTau6/UMD3.1 and bosTau8/UMD3.1.1) and is located between the first and second SNP of *IC*-*Hap4* (Fig. 2) [13]. This segment does not encompass any annotated protein coding gene but comprises a large number of repetitive elements (LINE and SINE). The nearest gene, *Bos taurus twist family BHLH transcription factor 2* (*TWIST2*; 118,630,617-118,690,527 bp), is located approximately 16 kb distal of *Belt*_*Multi6kb*_. Although the coverage curve suggested some kind of multiplication of this sequence, we were not able to identify the borders of this event since there were no reads that showed a common breakpoint for correctly mapped and mis-mapped bases, as is frequently seen in duplication events.

### Results of BAC re-sequencing

NGS identified an obviously large number of SNPs that were homozygous and different from the reference sequence, especially in the proximal third part of the *Belt*_*Multi6kb*_ region, in animals from all tested breeds (Fig. 2c), whether belted or not. Thus, to check the reliability of the reference sequence, BAC clones CH240-363B2 and CH240-104M22 were sequenced. It should be noted that the DNA used to construct the CH240 library originated from a male Hereford individual, i.e. a non-belted animal. The results clearly refuted the occurrence of assembly errors in the candidate region of the reference sequence, as demonstrated by the pairwise local alignment of the sequence of CH240-104M22 and the UMD3.1 reference sequence of the candidate region (chr3:118,608,000-118,614,000), which was performed using EMBOSS Water [43]. As shown in Additional file 2, a similarity of 99.9% between the re-sequenced BAC CH240-104M22 and the reference sequence including only five SNPs and no gap in 6001 aligned bases was found.

### Results of qPCR

**Fig. 3:**
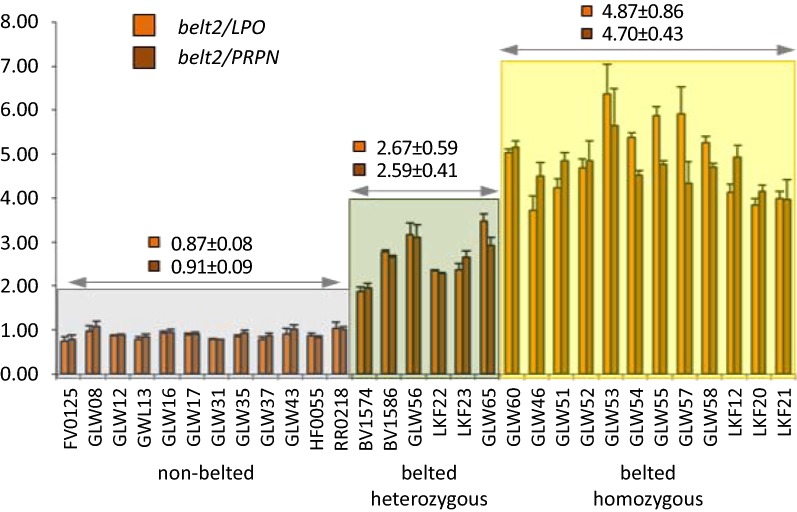
Relative copy numbers of the candidate region. The copy numbers for the amplicon belt2 within the candidate segment were compared to the copy numbers of the reference sites in *LPO* and *PRPN*, respectively. Animals with a proven non-belted phenotype (highlighted in grey) clearly differ from those with a presumably heterozygous belted (green) or homozygous belted (yellow) genotype. The mean values of copy number ratios ± standard deviation are indicated for each group. Sample ID correspond to breeds: FV: German Fleckvieh; HF: Holstein–Friesian; GLW: (Belted) Galloway; BV: GUV; LKF: DBE; RR: crossbred

**Fig. 4:**
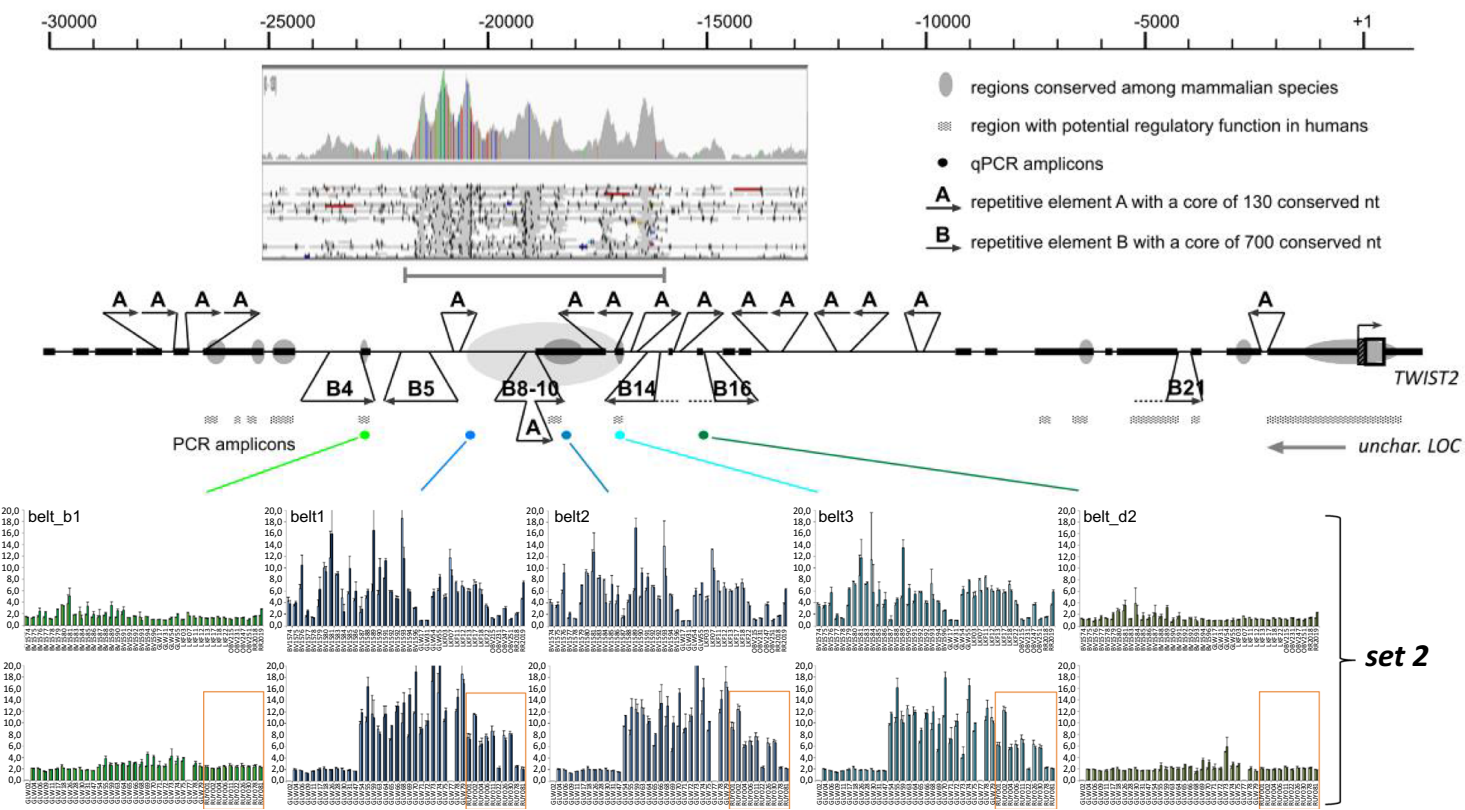
Bioinformatic examination of the candidate region. The upstream 30-kb region of the bovine *TWIST2* gene is indicated with the transcription start of the gene representing position +1 on the scale bar. Regions that resemble unique bovine sequences are represented by bold lines. Regions that appear to be conserved among mammalian species are marked by grey ellipses and regions that are predicted to have a regulatory function in humans are indicated by a dotted bar. Regions that resemble repetitive elements are indicated by thin lines. The multiple occurring repetitive elements A and B are highlighted with arrows indicating their approximate size and orientation. The candidate region that was predicted by an increased number of NGS reads (inset) is shown by a grey arrow with the localization of the amplicons used in the qPCR indicated by colored dots. The shown qPCR results represent the results of the second set (see “Results of qPCR”). The top row comprises animals from the Belted and Black Galloway (GLW), Braunvieh (new and old breeding type) and Gurtenvieh (BV or OBV) breeds, and crosses between Gurtenvieh and Pinzgauer (RR) and Dutch Belted (LKF). The bottom row comprises animals from the Belted and Black Galloway (GLW) and Russian Yakutian (RUY) breeds (the latter highlighted by orange boxes)

In order to elucidate why there was an increased number of reads mapping to *Belt*_*Multi6kb*_ in the NGS data, we determined the relative copy numbers in belted and non-belted animals by qPCR (Fig. 3). In a first set, we determined the relative copy numbers for 12 non-belted and 18 belted animals, with a heterozygous status predicted from haplotypes for six animals and a homozygous status for 12 animals of the latter group. All non-belted animals showed a relative copy number of approximately 1 for the qPCR amplicon belt2 (Figs. 3, 4) as well as for the amplicons belt1 and belt3 (Fig. 4). The copy number ratio between the reference sites in *LPO* and *PRPN* was also close to 1 (data not shown). Since cattle is a diploid species, a ratio of 1 represents copies from two chromosomes. The relative copy number ratio of the belt amplicon sites increased to more than 2 for animals that presumably carried the belted genotype in a heterozygous state and more than 4 in animals that were presumably homozygous belted. These results confirmed that *Belt*_*Multi6kb*_ most likely represents a quadruplication of the 6-kb candidate region. For all the animals studied, the phenotypes predicted from qPCR data agreed with those predicted from haplotype data. Interestingly, the qPCR also confirmed the haplotype-based prediction of heterozygous genotypes for two Dutch Belted individuals, which contradicts the initial assumption used for remapping (see section “Animal samples”) that all Dutch Belted animals are homozygous belted. The qPCR amplicons flanking the candidate region (belt_b1, belt_d2) showed copy number ratios close to 1 for all animals irrespective of the belted phenotype. Thus, the multiplication points could be restricted to the segments between belt_b1 and belt1 and between belt3 and belt_d2 (Fig. 4). In a second set, we checked another 70 animals that included, in addition to Belted Galloway, Dutch Belted and Gurtenvieh individuals, eight belted and two non-belted individuals from the Russian Yakutian breed. Interestingly, all but one belted Russian Yakutian individual also showed increased copy numbers for all three belt amplicon sites but not for the two flanking amplicons (Fig. 4).

To investigate the hypothesis that the size or physical appearance of a belt could be associated with multiplication events other than a quadruplication, we included five animals that showed a very unsymmetrical belt, a very thin or very broad belt, a belt that did not circle the body completely and a belt that contained colored spots. However, qPCR results showed that, for these animals, copy numbers were comparable to those for animals with a typical belt and, thus, did not support the hypothesis (data not shown).

### Results of targeted locus amplification (TLA)

Targeted locus amplification (TLA) was conducted in a belted and a non-belted GLW (Galloway) individual and confirmed the previous results of this study. Amplification of the candidate region and its surrounding regions showed that the genome coverage in the candidate region was 3 to 4.5 times higher in the genome of the belted Galloway individual than that of the non-belted Galloway individual (see Additional file 3). The start and end positions of the multiplication event were estimated to lie between 118,607,797 and 118,608,377 bp and between 118,613,882 and 118,614,044 bp, respectively. Based on the observed copy number and the fact that no breakpoint was detected, a multiplication event with homologous recombination at the sites of repetitive elements was hypothesized. This hypothesis is supported by the fact that the same LINE element (BovB) occurs at the beginning and the end of the candidate locus. The similarity between both BovB-sequences was clearly seen in the dotplot performed with Gepard 1.4 [44] (Fig. 2d), and a global pairwise alignment performed with EMBOSS Needle [45] (see Additional file 4) confirmed an identity of more than 80% between both elements.

### Results of nanopore sequencing

Whole-genome sequencing produced approximately 6.8 Gb of sequence of which about 98% could be mapped to the bovine genome. Read lengths extended up to 25 kb, but most of the reads were shorter than 5 kb. Mapping results in the candidate region for the belted trait showed five long reads that partially mapped to the end of the suspected repeat expansion and partially to its beginning (see Additional file 5a and b), thus confirming the head-to-tail repetition of the candidate region. An attempt to assemble the candidate region from long reads resulted in only a relatively short contig of 5 kb, but it showed the junction between the putatively amplified regions and thus confirmed the head-to-tail orientation. While the overall genome coverage was only about twofold, it was tenfold in the candidate region. Five reads showed the presence of a head-to-tail junction between the putative repeat units. A simple duplication would only show junction-spanning reads in the range of the average genome coverage (2 ×), thus the five split reads correspond approximately to three junctions, i.e. a fourfold repetition of the unit. The breakpoints of the repeated units were determined by the results of the assembly and the split alignment of junction-spanning reads at 118,608,362 and 118,614,132 bp. At position 118,614,132 (the breakpoint on the right side), five fragments showed a “soft-clip” from which a consensus sequence GGACCAGATGCTGATTTGTTTTC… could be derived (see Additional file 5c). This sequence is present at the beginning of the candidate region and thus defines the left breakpoint (at position 118,608,362). A formal confirmation of the quadruplication event would need a nanopore read that spans the entire amplified region. While this is technically possible, these reads are usually present only a small fraction of the whole sequencing run, and thus would require a high throughput flow cell that yields ~ 100 Gb of sequence from a single run. Such flow cells are currently under development.

## Discussion

The NGS, qPCR, TLA and nanopore sequencing results consistently showed an increase in the number of copies of a region between approximately 118,608,00 and 118,614,000 bp on BTA3 in belted animals compared to non-belted animals. Thus, our findings confirm the multiplication event that was previously also described by Awasthi Mishra et al. [15] as the most likely candidate for the belted phenotype in cattle. Interestingly, the qPCR, nanopore and TLA sequencing results suggest that the belted phenotype is probably not due to a simple duplication of the candidate region, but more likely to a quadruplication of this region. The molecular basis of such a process remains elusive, but a detailed analysis of the candidate region and its surrounding regions illustrates that this locus carries a considerable number of two types of repetitive elements referred to as “A” and “B” (Fig. 4). While the elements of A and B are not identical, the high degree of homology between them might be sufficient for homologous recombination, which, in the end, caused multiplication of the locus. The high density of repetitive elements within and around the candidate region also hampers the definition of the complete multiplied region so that the exact nucleotide sequence of *Belt*_*Multi6kb*_ remains elusive. In general, however, there is evidence that after a rare event of a duplication, the chance of getting higher orders of duplications at the same site is elevated based on asymmetric pairing and crossing-over [46].

### Exact extension and particularities of the candidate *belt* locus

Although the approximate position of the *Belt*_*Multi6kb*_ candidate locus is obvious (118,608,000–118,614,000 bp) and the putative breakpoints could be identified, the exact nature of the multiplication event is yet unknown. Most probably, a homologous recombination within the repetitive elements that are located on both sides of the multiplied region (Fig. 2d) led to a stepwise amplification of the region. Mapping of long nanopore reads defined the outer boundaries of the multiplication at 118,608,362 and 118,614,132 bp. However, the exact multiplicity and the inner breakpoints could not be determined due to the relatively low coverage and limited read length. The outer breakpoints do suggest that repetitive elements at the beginning and end of the *Belt*_*Multi6kb*_ region are involved. Interestingly, the annotated repetitive elements differ partly in their classification regarding long (LINE) and short interspersed nuclear elements (SINE) in bosTaurus6 and bosTaurus8 reference genomes, although an exemplary pairwise alignment shows that both sequences are identical (see Additional file 6). Interspersed nuclear elements are known to be involved in genome instability by generating copy number variants [47]. We hypothesize that a similar process has caused the multiplication event leading to the belted phenotype. For quadruplication, independent recurrences of this process could have occurred.

### *TWIST2* as the most likely candidate gene

The gene *TWIST2* represents the nearest protein coding sequence and is located approximately 16 kb downstream of the multiplication event. We agree with Awasthi Mishra et al. [15] that this gene is most likely affected by the multiplication event. Several reasons supporting this assumption are presented in Awasthi Mishra et al. [15].

Another indication that strengthens the assumption that *TWIST2* could be involved in the formation of the belted phenotype in cattle is the fact that a gene interaction network prepared with genemania [48] connects *TWIST2* with both *KIT* and *ADAMTS20*, which are the causal genes for belted phenotypes in pigs and mice (see Additional file 7). In addition to various indirect connections, there are also direct connections between all three genes (even if only predicted functional relationships).

### Hypotheses for different belt widths

Since the candidate variant for the belted phenotype is most likely a quadruplication, the question was raised whether animals with a smaller number of multiplication events, i.e. duplications or triplications, showed an imperfect or thin belt, and those with more than four duplications showed unusually broad belts. The assumption that copy numbers other than 1 (non-belted allele) and 4 (belted allele) may occur correlates with the results of Awasthi Mishra et al. who found varying copy numbers of 2 to 12 by digital droplet PCR [15]. To check this hypothesis, we included six animals with imperfect belts within the qPCR analysis. However, the qPCR results of the animals with imperfect belts showed comparable copy numbers to those of the typically belted animals and, thus, did not support this hypothesis. However, it should be noted that due to the small DNA concentrations (less than 10 pg/µL in some cases), the results of the second qPCR set clearly confirmed the separation of non-belted and belted animals (Fig. 4) but only hardly allowed a differentiation between homozygous and heterozygous belted animals. Consequently, based on the current data, a connection between belt width/appearance and copy number seems unlikely but cannot be excluded for certain.

### The belt in Russian Yakutian cattle

The qPCR results presented in this study strongly indicate that the belt phenotype of belted Russian Yakutian animals has the same or at least a very similar genetic cause than that of Gurtenvieh, Dutch Belted and Belted Galloway cattle since all but one belted Russian Yakutian individual also showed an elevated copy number for the amplicons belt1-3 but not for those outside of the candidate region. However, we were not able to check if the supposedly belted Russian Yakutian individual that did not carry the multiplication event was indeed belted or maybe piebald because no pictures were available and the animal was no longer available for a second phenotyping and sampling.

While breeding histories strongly indicate that the belt phenotype was introgressed into Dutch Belted from Gurtenvieh cattle and then into Belted Galloway from Dutch Belted cattle, a direct connection of one of these breeds to the Russian Yakutian breed (or the other way round) is not known to the authors. However, an indirect connection could be found, i.e. that cattle from the Netherlands were crossed with Kholmogory cattle in 1725 and, again, in small numbers during the years 1765 to 1898 [49]. Kholmogory bulls, in turn, were frequently crossed with Yakutian cattle in the years after 1929 [50]. Since Kholmogory cattle are a piebald breed, the belts may not be clearly visible with animals that have a large proportion of white coat, thus the belted phenotype may have been introduced into both Kholmogory and Russian Yakutian cattle unintentionally. In that case, we would expect belted Russian Yakutian cattle to have the Dutch Belted haplotype or at least a similar extended haplotype such as the Gurtenvieh, Dutch Belted and GLW breeds. However, as shown in Additional file 8, the haplotypes of the belted Russian Yakutian are, especially within *IC*-*Hap4*, much less extended and less uniform than in the European cattle breeds, which is an indication that the belted mutation has been segregating in the North-East Asian Turano cattle group for a much longer time. Thus, it could also be possible that the belt phenotype was introduced into European cattle breeds by some North-East Asian cattle or that the mutation already existed when the progenitors of the North-East Asian and European breeds spread. Future studies using IBD analysis of high-density SNP data for haplotypes flanking the belted mutation, or third-generation sequencing, or even more advanced methods that might be developed within the next years and that will hopefully be able to sequence the whole multiplication event in one piece, could finally clarify the question if the North-East Asian and European belted mutation events are completely identical or not.

## Conclusions

Our results confirm the findings by Awasthi Mishra et al. [15] in an independent animal set and indicate that a putative 6-kb quadruplication on BTA3, which is positioned between 118,608,362 and 118,614,132 bp and might affect its nearest gene *TWIST2*, is the most likely candidate causal variant for the belted phenotype in European and very likely even Siberian cattle. Moreover, this study highlights the complexity of this highly repetitive genomic region and demonstrates that, in particular, the beginning and end of the multiplication event are formed by highly similar sequences. Finally, the breakpoints defined here could enable a PCR-based test that would be specific of the junction generated by the multiplication event. However, the repetitive nature of the joined elements may prevent this obvious solution. Although this study adds important new information to the process of elucidating the belted coat color phenotype in cattle, several questions remain that need future investigations: (i) do multiplication events other than quadruplications exist and do the respective animals show plain-colored or belted phenotypes, (ii) are the multiplication events in European and North-East Asian Turano cattle identical or do they represent two independent similar mutations, and (iii) (in which way) does the multiplication event influence the putative candidate gene *TWIST2*.

## Additional files

**Additional file 1.** Inner candidate haplotypes detected by manual analysis of the extended confidence interval. This file shows the 60-SNP haplotypes of the extended candidate interval for all 110 animals that were used for remapping of the *belt* locus. SNPs that were excluded from the mapping procedure (MAF < 0.025) are marked with grey color in the first line. The black box indicates the 336-kb interval identified by Drögemüller et al. [13]. The first five haplotypes represent the most common and extended haplotypes for Belted Galloway (BGAhap1 and BGAhap2, shown in bright and dark blue), Dutch Belted (DBEhap, shown in beige) and Gurtenvieh (GUVhap1 and GUVhap2, shown in dark and bright green). Red boxes indicate common parts of these five haplotypes and represent the four inner candidate haplotypes (*IC*-*Hap*1-4, Table 3). The haplotypes of the animals that were used for remapping are shown below in the following order: (i) Belted Galloway, (ii) Dutch Belted, (iii) Gurtenvieh, (iv) a belted cross between Gurtenvieh and Pinzgauer cattle and (v) non-belted control animals.

**Additional file 2.** Alignment of CH240-104M22 and the reference sequence. Pairwise alignment of the BAC-clone CH240-104M22 with the bosTaurus6 reference sequence of the 6-kb candidate region showing almost complete identity.

**Additional file 3.** TLA results. The genomic region chr3:118,590,000–118,632,000 (bosTau8) is displayed. The arrows indicate the position of the primer sets used for TLA. For the belted animal (GLW54-2), an increased copy number (3 to 4.5 times) was detected in the region indicated by the red rectangle. The y-axis is limited to max. 1000X.

**Additional file 4.** Alignment of the repetitive elements at the beginning and end of the 6-kb candidate segment. Pairwise alignment of the reference sequences (BosTaurus8) of LINE BovB at the beginning of the 6-kb candidate segment and LINE BovB at the end of the segment. This file shows the huge level of similarity between the start and end of the candidate segment as is also obvious in Fig. 2d.

**Additional file 5.** Nanopore sequencing results. (a) Nanopore reads mapped (minimap2) to the belted candidate region are shown. The shaded parts of the reads highlight unmapped portions of the read that were mapped as a secondary alignment in a separate read. These secondary alignments are highlighted by blue borders. (b) Split-alignment of breakpoint-spanning read visualized by Ribbon [52]. The highlighted read (bold blue line) is shown as a zoom in the lower panel, showing that the beginning of the read is found at the end of the repeated region and the end is found at the beginning, thus illustrating the concatenation of the repeat units found in belted cattle. (c) Exact breakpoints were identified by inspection of the partially mapped reads. The right breakpoint at 118,614,132 bp shows that the unmapped portion of the split-aligned reads starts with a sequence that is located at 118,608,362 bp, which thus defines the left breakpoint.

**Additional file 6.** Alignment of the repetitive elements at the beginning of the 6-kb candidate segment according to bosTaurus6 and bosTaurus8. The pairwise alignment of the reference sequence of the SINE element ART2A (bosTau6) and the LINE element BovB (bosTau8) at the beginning of the 6-kb candidate segment shows that ART2A is part of BovB.

**Additional file 7.** Gene interaction network. This figure illustrates the interactions between *KIT* (causal for the belt in pigs), *ADAMTS20* (causal for the belt in mice) and *TWIST2* (most likely causal for the belt in cattle) in mice. Interaction line colors are as follows: orange: predicted functional relationship, red: physical interactions, purple: co-expression; grey: phenotype (based on mouse genome informatics) and blue: participation in the same reaction within a pathway.

**Additional file 8.** Haplotypes of the Russian Yakutian animals checked by qPCR. This file shows 42-SNP haplotypes of the 10 Russian Yakutian animals that were checked by qPCR. As in Additional file 1, SNPs that were excluded from the mapping procedure (MAF < 0.025) are marked in grey color in the first line, and the first five haplotypes represent the most common and extended haplotypes of the European breeds Belted Galloway (BGAhap1 and BGAhap2), Dutch Belted (DBEhap) and Gurtenvieh (GUVhap1 and GUVhap2). Red boxes again indicate common parts of these five haplotypes and represent the four inner candidate haplotypes (*IC*-*Hap*1-4, Table 3). Below these common haplotypes, the haplotypes of the 10 Siberian Russian Yakutian animals are grouped as follows: the first seven animals were belted according to phenotype and qPCR, the next animal was belted according to phenotype but non-belted in the qPCR, and the last two animals were non-belted according to phenotype and qPCR. Interestingly, the belted Russian Yakutian (RUY) animals do not share a common haplotype within *IC*-*Hap4,* which carries the candidate mutation *Belt*_*Multi6kb*_.
