# Supplementary material for: Remapping of the belted phenotype in cattle on BTA3 identifies a multiplication event as the candidate causal mutation
Source: Genet Sel Evol. 2018 Jul 6;50:36. doi: 10.1186/s12711-018-0407-9 (PMC6035435; doi:10.1186/s12711-018-0407-9)
Supplement: Supplementary file 2 — Additional file 2. Alignment of CH240-104M22 and the reference sequence. Pairwise alignment of the BAC-clone CH240-104M22 with the bosTaurus6 reference sequence of the 6-kb candidate region showing almost complete identity. [file 12711_2018_407_MOESM2_ESM.pdf]

```
#=======================================
#
# Aligned_sequences: 2
# 1: bosTau6_CR_rev
# 2: CH240-104M22
# Matrix: EDNAFULL
# Gap_penalty: 10.0
# Extend_penalty: 0.5
#
# Length: 6001
# Identity:    5996/6001 (99.9%)
# Similarity:  5996/6001 (99.9%)
# Gaps:           0/6001 ( 0.0%)
# Score: 29960.0
#
#
#=======================================

bosTau6_CR_re      1 CCTAGATAGCATATTCAAAAGCAGAGATATTACTTTGCCAACAAAGGTCC     50
                     ||||||||||||||||||||||||||||||||||||||||||||||||||
CH240-104M22   46656 CCTAGATAGCATATTCAAAAGCAGAGATATTACTTTGCCAACAAAGGTCC  46705

bosTau6_CR_re     51 ATCTAGTCAAGGCTGGTTTTTCCAGTGGTCATGTATGGATGTGAGAGTTG    100
                     ||||||||||||||||||||||||||||||||||||||||||||||||||
CH240-104M22   46706 ATCTAGTCAAGGCTGGTTTTTCCAGTGGTCATGTATGGATGTGAGAGTTG  46755

bosTau6_CR_re    101 GACTGTGAAGAAAACTGAGCACCGAAGAATTAATGCTTTTGAACTGTGGT    150
                     ||||||||||||||||||||||||||||||||||||||||||||||||||
CH240-104M22   46756 GACTGTGAAGAAAACTGAGCACCGAAGAATTAATGCTTTTGAACTGTGGT  46805

bosTau6_CR_re    151 GTTGGAGAAGACTCTTGAGACTCCCTTGGACTGCAAGGAGATGCAACCAG    200
                     ||||||||||||||||||||||||||||||||||||||||||||||||||
CH240-104M22   46806 GTTGGAGAAGACTCTTGAGACTCCCTTGGACTGCAAGGAGATGCAACCAG  46855

bosTau6_CR_re    201 TCCATTCTAAAGGAGATCAGCCCTGGGATTTCTTTGGAAGGAATAATGCT    250
                     ||||||||||||||||||||||||||||||||||||||||||||||||||
CH240-104M22   46856 TCCATTCTAAAGGAGATCAGCCCTGGGATTTCTTTGGAAGGAATAATGCT  46905

bosTau6_CR_re    251 AAAGCTGAAACTCCAGTACTTTGGCCACCTGATGTGAAGAGTTGACTCAT    300
                     ||||||||||||||||||||||||||||||||||||||||||||||||||
CH240-104M22   46906 AAAGCTGAAACTCCAGTACTTTGGCCACCTGATGTGAAGAGTTGACTCAT  46955

bosTau6_CR_re    301 TGGAAAAGACTCTGATGCTGGGAGGGATTGGGGGCAGGAGGAGAAGGGGA    350
                     ||||||||||||||||||||||||||||||||||||||||||||||||||
CH240-104M22   46956 TGGAAAAGACTCTGATGCTGGGAGGGATTGGGGGCAGGAGGAGAAGGGGA  47005

bosTau6_CR_re    351 CGACAGAGGATGAGATGGCTGGATGGCATCACCGACTCGATGGACGTGAG    400
                     ||||||||||||||||||||||||||||||||||||||||||||||||||
CH240-104M22   47006 CGACAGAGGATGAGATGGCTGGATGGCATCACCGACTCGATGGACGTGAG  47055

bosTau6_CR_re    401 TCTGAGTGAACTCCGGGAGTTGGTGATGGACAGGGAGGCCTGGTGTGTTG    450
                     ||||||||||||||||||||||||||||||||||||||||||||||||||
CH240-104M22   47056 TCTGAGTGAACTCCGGGAGTTGGTGATGGACAGGGAGGCCTGGTGTGTTG  47105

bosTau6_CR_re    451 CGATTCACGGGGTCGCAAAGAGTCGGACACGACTGAGCGACTGAACTGAA    500
                     ||||||||||||||||||||||||||||||||||||||||||||||||||
CH240-104M22   47106 CGATTCACGGGGTCGCAAAGAGTCGGACACGACTGAGCGACTGAACTGAA  47155

bosTau6_CR_re    501 CTGATGCTGACTACCTCTTTTTATGCTCTAACAAACATAGGGTAATCCGT    550
                     ||||||||||||||||||||||||||||||||||||||||||||||||||
CH240-104M22   47156 CTGATGCTGACTACCTCTTTTTATGCTCTAACAAACATAGGGTAATCCGT  47205

bosTau6_CR_re    551 GTGGCCCACCTCTCCTCACACTTGGGATTTAGTTTCTCTGTGGCCTTTTG    600
                     ||||||||||||||||||||||||||||||||||||||||||||||||||
CH240-104M22   47206 GTGGCCCACCTCTCCTCACACTTGGGATTTAGTTTCTCTGTGGCCTTTTG  47255

bosTau6_CR_re    601 AATTCAATTCACTAGGGACTAAAGGAACATGATGATCCTGTAGATTATCC    650
                     ||||||||||||||||||||||||||||||||||||||||||||||||||
```

```
CH240-104M22   47256 AATTCAATTCACTAGGGACTAAAGGAACATGATGATCCTGTAGATTATCC  47305

bosTau6_CR_re    651 AACTTCTCACTGTCAGAGTAAGAGTAGTGGTCCCCGGCAACTTTTTATAA    700
                     ||||||||||||||||||||||||||||||||||||||||||||||||||
CH240-104M22   47306 AACTTCTCACTGTCAGAGTAAGAGTAGTGGTCCCCGGCAACTTTTTATAA  47355

bosTau6_CR_re    701 CCCAAGTAGAAACAGTTCGTGCAAGCTCAGTTGCCCCATCGCTCAGTCGT    750
                     ||||||||||||||||||||||||||||||||||||||||||||||||||
CH240-104M22   47356 CCCAAGTAGAAACAGTTCGTGCAAGCTCAGTTGCCCCATCGCTCAGTCGT  47405

bosTau6_CR_re    751 GTCTGATTCTTTGCAACCTACGGACTGTAGCCCGGCAGACTCCTCTCTCC    800
                     ||||||||||||||||||||||||||||||||||||||||||||||||||
CH240-104M22   47406 GTCTGATTCTTTGCAACCTACGGACTGTAGCCCGGCAGACTCCTCTCTCC  47455

bosTau6_CR_re    801 ATGGAACTTTCCAGACAAGAATACTGAAGTGAGTTGCCACGTCCTATTCC    850
                     |||||||||||||||||||||||||||||||||||||||.||||||||||
CH240-104M22   47456 ATGGAACTTTCCAGACAAGAATACTGAAGTGAGTTGCCATGTCCTATTCC  47505

bosTau6_CR_re    851 AGGGGATCTTCCTGATCCAGGAATCAAACTCACAACTCTTGGGTCTCCTG    900
                     ||||||||||||||||||||||||||||||||||||||||||||||||||
CH240-104M22   47506 AGGGGATCTTCCTGATCCAGGAATCAAACTCACAACTCTTGGGTCTCCTG  47555

bosTau6_CR_re    901 AGTCTCCAGCACTGGCCGGCAGATTCTTTACCCCTGCGTCTCCTGGGAAG    950
                     ||||||||||||||||||||||||||||||||||||||||||||||||||
CH240-104M22   47556 AGTCTCCAGCACTGGCCGGCAGATTCTTTACCCCTGCGTCTCCTGGGAAG  47605

bosTau6_CR_re    951 CCGCAGATGGAAGCAGATTCCACTGAAAGCTCTCCCCAGCCATGTCTCAC   1000
                     ||||||||||||||||||||||||||||||||||||||||||||||||||
CH240-104M22   47606 CCGCAGATGGAAGCAGATTCCACTGAAAGCTCTCCCCAGCCATGTCTCAC  47655

bosTau6_CR_re   1001 CGCCACTGTCCCCCTGCTGGTCACTGTTATAGGCATACATGAGACATGCC   1050
                     ||||||||||||||||||||||||||||||||||||||||||||||||||
CH240-104M22   47656 CGCCACTGTCCCCCTGCTGGTCACTGTTATAGGCATACATGAGACATGCC  47705

bosTau6_CR_re   1051 GTGCGGTGGCCTCACCCATTCATGGCTGTTTGTGGTCAACAGAAGCATCT   1100
                     ||||||||||||||||||||||||||||||||||||||||||||||||||
CH240-104M22   47706 GTGCGGTGGCCTCACCCATTCATGGCTGTTTGTGGTCAACAGAAGCATCT  47755

bosTau6_CR_re   1101 AGCTTGCCTTTTACATCACTTGTTGCTGCTGCTGCTGCTGCTAAGTCACT   1150
                     ||||||||||||||||||||||||||||||||||||||||||||||||||
CH240-104M22   47756 AGCTTGCCTTTTACATCACTTGTTGCTGCTGCTGCTGCTGCTAAGTCACT  47805

bosTau6_CR_re   1151 TCAGTCGTGTCCGACTCTGTGTAACCCCAGAGACGGCAGCCCACCAGGCT   1200
                     ||||||||||||||||||||||||||||||||||||||||||||||||||
CH240-104M22   47806 TCAGTCGTGTCCGACTCTGTGTAACCCCAGAGACGGCAGCCCACCAGGCT  47855

bosTau6_CR_re   1201 TCCCTGTCCCTGGGATTCTCCAGGCAAGAACACTGGAGTGGGTTGCCATT   1250
                     ||||||||||||||||||||||||||||||||||||||||||||||||||
CH240-104M22   47856 TCCCTGTCCCTGGGATTCTCCAGGCAAGAACACTGGAGTGGGTTGCCATT  47905

bosTau6_CR_re   1251 TCCTTCTCCAATGCATGAAAGTGAAAAGTAAAAGTGAAGTCACTCAGTTG   1300
                     ||||||||||||||||||||||||||||||||||||||||||||||||||
CH240-104M22   47906 TCCTTCTCCAATGCATGAAAGTGAAAAGTAAAAGTGAAGTCACTCAGTTG  47955

bosTau6_CR_re   1301 TGTCCAACTCTTAGCGACCCCATGGACTGCAGCCTACCAGGCTCCTCCAT   1350
                     ||||||||||||||||||||||||||||||||||||||||||||||||||
CH240-104M22   47956 TGTCCAACTCTTAGCGACCCCATGGACTGCAGCCTACCAGGCTCCTCCAT  48005

bosTau6_CR_re   1351 CCATGGGATTTTCTAGGCAACAGTACTGGAGTGGGGTGCCATTGCCTTCT   1400
                     ||||||||||||||||||||||||||||||||||||||||||||||||||
CH240-104M22   48006 CCATGGGATTTTCTAGGCAACAGTACTGGAGTGGGGTGCCATTGCCTTCT  48055

bosTau6_CR_re   1401 CCACATCACTTGTTACCAGAGCTTATATTCAACTTGCATCTCATGAGTCT   1450
                     ||||||||||||||||||||||||||||||||||||||||||||||||||
CH240-104M22   48056 CCACATCACTTGTTACCAGAGCTTATATTCAACTTGCATCTCATGAGTCT  48105

bosTau6_CR_re   1451 CTTTGTCAACGTGATTTTGTTAGTCTTCCAAGGCTTAGGGGAAACTAATT   1500
                     ||||||||||||||||||||||||||||||||||||||||||||||||||
```

```
CH240-104M22   48106 CTTTGTCAACGTGATTTTGTTAGTCTTCCAAGGCTTAGGGGAAACTAATT  48155

bosTau6_CR_re   1501 TGTAAATTTTCAAAGGTGGGAGCTCTCCAAGTGAGAGTTCTTTCCCGTGT   1550
                     ||||||||||||||||||||||||||||||||||||||||||||||||||
CH240-104M22   48156 TGTAAATTTTCAAAGGTGGGAGCTCTCCAAGTGAGAGTTCTTTCCCGTGT  48205

bosTau6_CR_re   1551 GAAATCTAGTATCAGGAAGTGCACTGTATCCACGCACCCCTCGTCCTATC   1600
                     ||||||||||||||||||||||||||||||||||||||||||||||||||
CH240-104M22   48206 GAAATCTAGTATCAGGAAGTGCACTGTATCCACGCACCCCTCGTCCTATC  48255

bosTau6_CR_re   1601 CAGGACCCAGATGGATGAGTCGTGTCTCTCTCTGCCTGTGTCTGCCCATC   1650
                     ||||||||||||||||||||||||||||||||||||||||||||||||||
CH240-104M22   48256 CAGGACCCAGATGGATGAGTCGTGTCTCTCTCTGCCTGTGTCTGCCCATC  48305

bosTau6_CR_re   1651 TCTGACTCAGATGGGCACATCCAGAGAGGTCCCTAGTCTCTGCCTTCCCC   1700
                     ||||||||||||||||||||||||||||||||||||||||||||||||||
CH240-104M22   48306 TCTGACTCAGATGGGCACATCCAGAGAGGTCCCTAGTCTCTGCCTTCCCC  48355

bosTau6_CR_re   1701 CTTCCACCCAGAACACTCATCCGGGGCTACCACCTCGCAGGAGGTGTGAA   1750
                     ||||||||||||||||||||||||||||||||||||||||||||||||||
CH240-104M22   48356 CTTCCACCCAGAACACTCATCCGGGGCTACCACCTCGCAGGAGGTGTGAA  48405

bosTau6_CR_re   1751 TCACGGATCCCAGTGCGACAATTAGAACACTCCCGTTTGCTCTCAGAAGT   1800
                     ||||||||||||||||||||||||||||||||||||||||||||||||||
CH240-104M22   48406 TCACGGATCCCAGTGCGACAATTAGAACACTCCCGTTTGCTCTCAGAAGT  48455

bosTau6_CR_re   1801 GTCACTTTTTAGACAATATATTAAGCAGGCACCCTCGTCCTCAGGGATCA   1850
                     ||||||||||||||||||||||||||||||||||||||||||||||||||
CH240-104M22   48456 GTCACTTTTTAGACAATATATTAAGCAGGCACCCTCGTCCTCAGGGATCA  48505

bosTau6_CR_re   1851 GGTGGCCCCACTCAGCCGGAACGCCCTGGTGCTCAGTGCGTCTTGCCCTT   1900
                     ||||||||||||||||||||||||||||||||||||||||||||||||||
CH240-104M22   48506 GGTGGCCCCACTCAGCCGGAACGCCCTGGTGCTCAGTGCGTCTTGCCCTT  48555

bosTau6_CR_re   1901 GAACTGCTCCTGGGTTCTCCCAACCCCCAGCCTGTTGCTTGGTCCCATTG   1950
                     ||||||||||||||||||||||||||||||||||||||||||||||||||
CH240-104M22   48556 GAACTGCTCCTGGGTTCTCCCAACCCCCAGCCTGTTGCTTGGTCCCATTG  48605

bosTau6_CR_re   1951 TCACCCTGAGTGATGACCGCTCAGGGCAGACCCTCACTCTTCTTGGCCAT   2000
                     ||||||||||||||||||||||||||||||||||||||||||||||||||
CH240-104M22   48606 TCACCCTGAGTGATGACCGCTCAGGGCAGACCCTCACTCTTCTTGGCCAT  48655

bosTau6_CR_re   2001 GACACTTCTTTTGGAGGCAGCCTTGGGGAAGGGTCCTATTTGTTCCCCTC   2050
                     ||||||||||||||||||||||||||||||||||||||||||||||||||
CH240-104M22   48656 GACACTTCTTTTGGAGGCAGCCTTGGGGAAGGGTCCTATTTGTTCCCCTC  48705

bosTau6_CR_re   2051 TGCCTCGAGGTCCCTTCCCAGGGGCTGACTCAAGTGACCCAGGCTTCTCC   2100
                     ||||||||||||||||||||||||||||||||||||||||||||||||||
CH240-104M22   48706 TGCCTCGAGGTCCCTTCCCAGGGGCTGACTCAAGTGACCCAGGCTTCTCC  48755

bosTau6_CR_re   2101 TGCACAGGGACAGGGTTGGCTCAGCACTGCCATGATGAATCCAGAGCCCA   2150
                     ||||||||||||||||||||||||||||||||||||||||||||||||||
CH240-104M22   48756 TGCACAGGGACAGGGTTGGCTCAGCACTGCCATGATGAATCCAGAGCCCA  48805

bosTau6_CR_re   2151 GAGAGGCAGAACTCTCACGTGCAGAGTCTGCATTGAGTGAGACAGCTGGC   2200
                     ||||||||||||||||||||||||||||||||||||||||||||||||||
CH240-104M22   48806 GAGAGGCAGAACTCTCACGTGCAGAGTCTGCATTGAGTGAGACAGCTGGC  48855

bosTau6_CR_re   2201 CCACACATTTGTGGATTTATCCTGAAATCCAAGCTTGTCGGATGCTGAGG   2250
                     ||||||||||||||||||||||||||||||||||||||||||||||||||
CH240-104M22   48856 CCACACATTTGTGGATTTATCCTGAAATCCAAGCTTGTCGGATGCTGAGG  48905

bosTau6_CR_re   2251 GCGGCTCTGAGCTGGGGATCTGTACAGACCCAGGAGCCATTAAGCATCTT   2300
                     ||||||||||||||||||||||||||||||||||||||||||||||||||
CH240-104M22   48906 GCGGCTCTGAGCTGGGGATCTGTACAGACCCAGGAGCCATTAAGCATCTT  48955

bosTau6_CR_re   2301 GCCTTCTTTCCGGATGAACAGAGAGAGGCGCCTGATGCTAGTCACATGGC   2350
                     ||||||||||||||||||||||||||||||||||||||||||||||||||
```

```
CH240-104M22   48956 GCCTTCTTTCCGGATGAACAGAGAGAGGCGCCTGATGCTAGTCACATGGC  49005

bosTau6_CR_re   2351 AACTCACACTCATCCTCTGGCACGTTTCCTCTCTTCGTTCTGACGTTCCT   2400
                     ||||||||||||||||||||||||||||||||||||||||||||||||||
CH240-104M22   49006 AACTCACACTCATCCTCTGGCACGTTTCCTCTCTTCGTTCTGACGTTCCT  49055

bosTau6_CR_re   2401 TATCTTTTGGGTCATAAAGAGATTTCATATGATGTACATTTTCCTTTGAG   2450
                     ||||||||||||||||||||||||||||||||||||||||||||||||||
CH240-104M22   49056 TATCTTTTGGGTCATAAAGAGATTTCATATGATGTACATTTTCCTTTGAG  49105

bosTau6_CR_re   2451 TCTTTCTCCTTTGTTCAGAGGAGCTGTCTCCCCCTCCTCTTCTCCATAGG   2500
                     ||||||||||||||||||||||||||||||||||||||||||||||||||
CH240-104M22   49106 TCTTTCTCCTTTGTTCAGAGGAGCTGTCTCCCCCTCCTCTTCTCCATAGG  49155

bosTau6_CR_re   2501 GAAGGGAGCCCCCGAGCTCTGGACTTTTGGAAGGACATCCTATCATATTA   2550
                     ||||||||||||||||||||||||||||||||||||||||||||||||||
CH240-104M22   49156 GAAGGGAGCCCCCGAGCTCTGGACTTTTGGAAGGACATCCTATCATATTA  49205

bosTau6_CR_re   2551 GAGCATGCACACACACAACCTTTGATCCTTTCTCTGACACATGGTGCATT   2600
                     ||||||||||||||||||||||||||||||||||||||||||||||||||
CH240-104M22   49206 GAGCATGCACACACACAACCTTTGATCCTTTCTCTGACACATGGTGCATT  49255

bosTau6_CR_re   2601 GATCATCCTTCACAGACTTGCATTGTCTGTGGCTTACATCATGAAGCGTG   2650
                     ||||||||||||||||||||||||||||||||||||||||||||||||||
CH240-104M22   49256 GATCATCCTTCACAGACTTGCATTGTCTGTGGCTTACATCATGAAGCGTG  49305

bosTau6_CR_re   2651 GTCCTGTGAAGAGACTGGATCTGAAGAACACATGAGACAGAGAATGCGTA   2700
                     ||||||||||||||||||||||||||||||||||||||||||||||||||
CH240-104M22   49306 GTCCTGTGAAGAGACTGGATCTGAAGAACACATGAGACAGAGAATGCGTA  49355

bosTau6_CR_re   2701 TTGTCAGATTGATTTAAACTTAGAGGAATCAGCAGGAAATTGTTGGCATC   2750
                     ||||||||||||||||||||||||||||||||||||||||||||||||||
CH240-104M22   49356 TTGTCAGATTGATTTAAACTTAGAGGAATCAGCAGGAAATTGTTGGCATC  49405

bosTau6_CR_re   2751 ATGAATCATGGGTGAAAGATCTTTGCATCACAGGCAAAATGCTTCATTCC   2800
                     ||||||||||||||||||||||||||||||||||||||||||||||||||
CH240-104M22   49406 ATGAATCATGGGTGAAAGATCTTTGCATCACAGGCAAAATGCTTCATTCC  49455

bosTau6_CR_re   2801 AGACTCTGACGGTCTTACCCTGATGGCTCCTTCCAGTAAGGACTGCCCCT   2850
                     ||||||||||||||||||||||||||||||||||||||||||||||||||
CH240-104M22   49456 AGACTCTGACGGTCTTACCCTGATGGCTCCTTCCAGTAAGGACTGCCCCT  49505

bosTau6_CR_re   2851 TGGAAAGCATCACGAAAAATGAAAGGGTACTTTCCCAATTCTTTTATTTT   2900
                     ||||||||||||||||||||||||||||||||||||||||||||||||||
CH240-104M22   49506 TGGAAAGCATCACGAAAAATGAAAGGGTACTTTCCCAATTCTTTTATTTT  49555

bosTau6_CR_re   2901 TTCAAGTTTATTTTTAGAGCAGTTTTAGGTTCACAATAAAATAGAGAGGA   2950
                     ||||||||||||||||||||||||||||||||||||||||||||||||||
CH240-104M22   49556 TTCAAGTTTATTTTTAGAGCAGTTTTAGGTTCACAATAAAATAGAGAGGA  49605

bosTau6_CR_re   2951 AGAGAATTTCCACGTACATTTTGCCCCCCACCCCAAGCACAGCCTCTCCC   3000
                     ||||||||||||||||||||||||||||||||||||||||||||||||||
CH240-104M22   49606 AGAGAATTTCCACGTACATTTTGCCCCCCACCCCAAGCACAGCCTCTCCC  49655

bosTau6_CR_re   3001 CAATCCACAGCCCCCACCGGAGGGTGGTGCATGTGTTACAGCTGATGGAC   3050
                     ||||||||||||||||||||||||||||||||||||||||||||||||||
CH240-104M22   49656 CAATCCACAGCCCCCACCGGAGGGTGGTGCATGTGTTACAGCTGATGGAC  49705

bosTau6_CR_re   3051 CCATGCTTTGTGTGGTTGTTCAGTCACTCAGTCATGTCCGACTCTTTGCG   3100
                     ||||||||||||||||||||||||||||||||||||||||||||||||||
CH240-104M22   49706 CCATGCTTTGTGTGGTTGTTCAGTCACTCAGTCATGTCCGACTCTTTGCG  49755

bosTau6_CR_re   3101 ACCCCATGAATGGCAGCACGCCAGGCCTCCCTGTCCATCACCAGCTCCCA   3150
                     ||||||||||||||||||||||||||||||||||||||||||||||||||
CH240-104M22   49756 ACCCCATGAATGGCAGCACGCCAGGCCTCCCTGTCCATCACCAGCTCCCA  49805

bosTau6_CR_re   3151 GAGTTCACTCAGACTCACGTCCATCGAGTCAGTGATGCCATCCAGCCATC   3200
                     ||||||||||||||||||||||||||||||||||||||||||||||||||
```

```
CH240-104M22   49806 GAGTTCACTCAGACTCACGTCCATCGAGTCAGTGATGCCATCCAGCCATC  49855

bosTau6_CR_re   3201 TCATCCTCTGTCACCCCCTTTTCCTCCTGTCCCCCAATCCCTCCCAGCAT  3250
                     ||||||||||||||||||||||||||||||||||||||||||||||||||
CH240-104M22   49856 TCATCCTCTGTCACCCCCTTTTCCTCCTGTCCCCCAATCCCTCCCAGCAT  49905

bosTau6_CR_re   3251 CAGTCTTTTCCAGTGAGTCAGCTCTTCACATCAGGTGGCCAAAGGATTGG  3300
                     ||||||||||||||||||||||||||||||||||||||||||||||||||
CH240-104M22   49906 CAGTCTTTTCCAGTGAGTCAGCTCTTCACATCAGGTGGCCAAAGGATTGG  49955

bosTau6_CR_re   3301 AGCTTCAGCTTCAGCATCAGTCCTTCCAATGAATATTCAGGACTGATTTC  3350
                     ||||||||||||||||||||||||||||||||||||||||||||||||||
CH240-104M22   49956 AGCTTCAGCTTCAGCATCAGTCCTTCCAATGAATATTCAGGACTGATTTC  50005

bosTau6_CR_re   3351 CATTAGGATTGACTGGTTTGATCTTCTTGCCATTGAAGGGACTCTCAAGA  3400
                     ||||||||||||||||||||||||||||||||||||||||||||||||||
CH240-104M22   50006 CATTAGGATTGACTGGTTTGATCTTCTTGCCATTGAAGGGACTCTCAAGA  50055

bosTau6_CR_re   3401 GTCTTCTCCAACACCACAGTTCAAAAGCATCAATTCTTCACCACTCAGCC  3450
                     ||||||||||||||||||||||||||||||||||||||||||||||||||
CH240-104M22   50056 GTCTTCTCCAACACCACAGTTCAAAAGCATCAATTCTTCACCACTCAGCC  50105

bosTau6_CR_re   3451 TTGTTTATGGTTCAACTCTCACATCCATACACGACTATTGGAAAAACCAT  3500
                     ||||||||||||||||||||||||||||||||||||||||||||||||||
CH240-104M22   50106 TTGTTTATGGTTCAACTCTCACATCCATACACGACTATTGGAAAAACCAT  50155

bosTau6_CR_re   3501 AGCTTTGGCTATACAGCTTCCCTGGTAGCTCAGTTGGTAAAAGAACCTGC  3550
                     ||||||||||||||||||||||||||||||||||||||||||||||||||
CH240-104M22   50156 AGCTTTGGCTATACAGCTTCCCTGGTAGCTCAGTTGGTAAAAGAACCTGC  50205

bosTau6_CR_re   3551 CTGCAGTGCAGGAGACCTGGGTTTGATCCCTGGGTCGGGAAGATGCCCTG  3600
                     ||||||||||||||||||||||||||||||||||||||||||||||||||
CH240-104M22   50206 CTGCAGTGCAGGAGACCTGGGTTTGATCCCTGGGTCGGGAAGATGCCCTG  50255

bosTau6_CR_re   3601 GAGGAGGAAATGGCAACCCACTCCAGTATTCTTGCCTGGAGAACCCCATG  3650
                     ||||||||||||||||||||||||||||||||||||||||||||||||||
CH240-104M22   50256 GAGGAGGAAATGGCAACCCACTCCAGTATTCTTGCCTGGAGAACCCCATG  50305

bosTau6_CR_re   3651 GACAGAGGAGCCTGGTGAGTTACAGTCCATCTAGAGGCGAGAGTCAGATG  3700
                     ||||||||||||||||||||||||||||||||||||||||||||||||||
CH240-104M22   50306 GACAGAGGAGCCTGGTGAGTTACAGTCCATCTAGAGGCGAGAGTCAGATG  50355

bosTau6_CR_re   3701 CGACTGAGTGACTGACCCACCAATGACTATACAGACCTTTGTCAGCAAAG  3750
                     ||||||||||||||||||||||||||||||||||||||||||||||||||
CH240-104M22   50356 CGACTGAGTGACTGACCCACCAATGACTATACAGACCTTTGTCAGCAAAG  50405

bosTau6_CR_re   3751 TGATGTCCCTGCTTTTGAATATGCTGTCTACGTTTGTCATAGCTTTCTGT  3800
                     ||||||||||||||||||||||||||||||||||||||||||||||||||
CH240-104M22   50406 TGATGTCCCTGCTTTTGAATATGCTGTCTACGTTTGTCATAGCTTTCTGT  50455

bosTau6_CR_re   3801 CTAAGGAGCAAGCGTCTCTTAATTTCATGGCTGCAGACACCATCTTCAGT  3850
                     ||||||||||||||||||||||||||||||||||||||||||||||||||
CH240-104M22   50456 CTAAGGAGCAAGCGTCTCTTAATTTCATGGCTGCAGACACCATCTTCAGT  50505

bosTau6_CR_re   3851 GGACTTATGCTGGTACATCGCAATTACCCAAGGTCCATACTTTATATAAG  3900
                     ||||||||||||||||||||||||||||||||||||||||||||||||||
CH240-104M22   50506 GGACTTATGCTGGTACATCGCAATTACCCAAGGTCCATACTTTATATAAG  50555

bosTau6_CR_re   3901 GCTCACTTTTGGTGTTGCGTATTCTGTGTGTTTGGACAAGTATCATCATT  3950
                     ||||||||||||||||||||||||||||||||||||||||||||||||||
CH240-104M22   50556 GCTCACTTTTGGTGTTGCGTATTCTGTGTGTTTGGACAAGTATCATCATT  50605

bosTau6_CR_re   3951 ATATACATGATATCATACAAAGCATTTTCACTGCCCTAAAGATTATCTGT  4000
                     ||||||||||||||||||||||||||||||||||||||||||||||||||
CH240-104M22   50606 ATATACATGATATCATACAAAGCATTTTCACTGCCCTAAAGATTATCTGT  50655

bosTau6_CR_re   4001 GTTCCTCCCATTCATGCCTCCTTGACCCACTGGCAGCTGCTGGTCCTTTA  4050
                     ||||||||||||||||||||||||||||||||||||||||||||||||||
```

```
CH240-104M22   50656 GTTCCTCCCATTCATGCCTCCTTGACCCACTGGCAGCTGCTGGTCCTTTA  50705

bosTau6_CR_re   4051 ATCATCTCCACAGTTTTGCTTTTTCCAGAACAACATAAAGTTGGAATCCT   4100
                     ||||||||||||||||||||||||||||||||||||||||||||||||||
CH240-104M22   50706 ATCATCTCCACAGTTTTGCTTTTTCCAGAACAACATAAAGTTGGAATCCT  50755

bosTau6_CR_re   4101 ACAGTGCGTAGTTTTCTCAGATTAACTTCCTCTGTTTAGTAATAAGCATT   4150
                     ||||||||||||||||||||||||||||||||||||||||||||||||||
CH240-104M22   50756 ACAGTGCGTAGTTTTCTCAGATTAACTTCCTCTGTTTAGTAATAAGCATT  50805

bosTau6_CR_re   4151 TAAGTTTCCTCGATGTCACTTCGTTGTTTGAAGGCTCATTTCTTTTTAGA   4200
                     ||||||||||||||||||||||||||||||||||||||||||||||||||
CH240-104M22   50806 TAAGTTTCCTCGATGTCACTTCGTTGTTTGAAGGCTCATTTCTTTTTAGA  50855

bosTau6_CR_re   4201 ACTGAATAATATTTCATTGTCTGGATGTACCCTCGTGTATTTATCCATTC   4250
                     ||||||||||||||||||||||||||||||||||||||||||||||||||
CH240-104M22   50856 ACTGAATAATATTTCATTGTCTGGATGTACCCTCGTGTATTTATCCATTC  50905

bosTau6_CR_re   4251 ACCTACTAAGGGACATTTTGACAATTATGAATAAAGCTGCTATACATATC   4300
                     ||||||||||||||||||||||||||||||||||||||||||||||||||
CH240-104M22   50906 ACCTACTAAGGGACATTTTGACAATTATGAATAAAGCTGCTATACATATC  50955

bosTau6_CR_re   4301 TGTGCTAATTTTCATCTGAACGTGAGTTTTCAACTCTGAGTAAATATCAA   4350
                     ||||||||||||||||||||||||||||||||||||||||||||||||||
CH240-104M22   50956 TGTGCTAATTTTCATCTGAACGTGAGTTTTCAACTCTGAGTAAATATCAA  51005

bosTau6_CR_re   4351 GGCGAATGTTGGCTGAATCCTATGGCTAGAGTATGCTTAGGTTTGTAAGA   4400
                     ||||||||||||||||||||||||||||||||||||||||||||||||||
CH240-104M22   51006 GGCGAATGTTGGCTGAATCCTATGGCTAGAGTATGCTTAGGTTTGTAAGA  51055

bosTau6_CR_re   4401 AAATAAACTGTCTTCAAGGTGGCTGTATCACTCTGCATTCCCACCAGCAG   4450
                     ||||||||||||||||||||||||||||||||||||||||||||||||||
CH240-104M22   51056 AAATAAACTGTCTTCAAGGTGGCTGTATCACTCTGCATTCCCACCAGCAG  51105

bosTau6_CR_re   4451 TAAATCAGAGTTCTTGCTGTTGCGTATTCTCGGCCACATTTGCTGTTGCC   4500
                     ||||||||||||||||||||||||||||||||||||||||||||||||||
CH240-104M22   51106 TAAATCAGAGTTCTTGCTGTTGCGTATTCTCGGCCACATTTGCTGTTGCC  51155

bosTau6_CR_re   4501 AGGGTTCAGATTTTGCCTTTCTGATTGCTATGTGGTAGTATCTCGTTGTT   4550
                     ||||||||||||||||||||||||||||||||||||||||||||||||||
CH240-104M22   51156 AGGGTTCAGATTTTGCCTTTCTGATTGCTATGTGGTAGTATCTCGTTGTT  51205

bosTau6_CR_re   4551 TGCATTTACCTGGTGGCCTATGATGGGGGTATCTCTACTTACTTGTCATC   4600
                     ||||||||||||||||||||||||||||||||||||||||||||||||||
CH240-104M22   51206 TGCATTTACCTGGTGGCCTATGATGGGGGTATCTCTACTTACTTGTCATC  51255

bosTau6_CR_re   4601 AGCACATCTTTAGTGAGGTGTCTGTTAACATCTTAGTCCATTTTTTAATT   4650
                     ||||||||||||||||||||||||||||||||||||||||||||||||||
CH240-104M22   51256 AGCACATCTTTAGTGAGGTGTCTGTTAACATCTTAGTCCATTTTTTAATT  51305

bosTau6_CR_re   4651 AGGCTGACTGCGTTTTTAGTGCTGCATTTTAAGAGTTCTTTGTACATTTT   4700
                     ||||||||||||||||||||||||||||||||||||||||||||||||||
CH240-104M22   51306 AGGCTGACTGCGTTTTTAGTGCTGCATTTTAAGAGTTCTTTGTACATTTT  51355

bosTau6_CR_re   4701 GGAAAACAATCCTGTATGTTTCTTTTGCAAGTATTTTCCTCTTGTCCACG   4750
                     ||||||||||||||||||||||||||||||||||||||||||||||||||
CH240-104M22   51356 GGAAAACAATCCTGTATGTTTCTTTTGCAAGTATTTTCCTCTTGTCCACG  51405

bosTau6_CR_re   4751 GTTTGTCTTCTCATTCCTTTAACGTTGTCCTTGAGTTCCCCTGGTGGCTC   4800
                     ||||||||||||||||||||||||||||||||||||||||||||||||||
CH240-104M22   51406 GTTTGTCTTCTCATTCCTTTAACGTTGTCCTTGAGTTCCCCTGGTGGCTC  51455

bosTau6_CR_re   4801 AGCCGGCAGAGAATCCGCCTGCAATGCAGCAAACCCAGCTTCAATCCCTG   4850
                     ||||||||||||||||||||||||||||||||||||||||||||||||||
CH240-104M22   51456 AGCCGGCAGAGAATCCGCCTGCAATGCAGCAAACCCAGCTTCAATCCCTG  51505

bosTau6_CR_re   4851 CATTGAAGGTCTCCTGGAGAAGGGAATGGCAGCCCACTCCAGTATTCTTG   4900
                     ||||||||||||||||||||||||||||||||||||||||||||||||||
```

```
CH240-104M22   51506 CATTGAAGGTCTCCTGGAGAAGGGAATGGCAGCCCACTCCAGTATTCTTG  51555

bosTau6_CR_re   4901 CCTGGAGAACCCCATAGACCGAAGAGCCTGGTGGGTTACAGTCCATGGGG   4950
                     ||||||||||||||||||||||||||||||||||||||||||||||||||
CH240-104M22   51556 CCTGGAGAACCCCATAGACCGAAGAGCCTGGTGGGTTACAGTCCATGGGG  51605

bosTau6_CR_re   4951 TCACAAAGAGTCAGACACAACCAAGTGACTAACACACTTGCAGGGCTGAA   5000
                     ||||||||||||||||||||||||||||||||||||||||||||||||||
CH240-104M22   51606 TCACAAAGAGTCAGACACAACCAAGTGACTAACACACTTGCAGGGCTGAA  51655

bosTau6_CR_re   5001 GTTTTTAATTTTAATGAAGTCCAGCGTGTCAATTCTTTCTTTCATGAATG   5050
                     ||||||||||||||||||||||||||||||||||||||||||||||||||
CH240-104M22   51656 GTTTTTAATTTTAATGAAGTCCAGCGTGTCAATTCTTTCTTTCATGAATG  51705

bosTau6_CR_re   5051 GTGCTTTTAGTATCGTACCTAAAAAGTCATCACCGTACCTAAGGTCATCT   5100
                     ||||||||||||||||||||||||||||||||||||||||||||||||||
CH240-104M22   51706 GTGCTTTTAGTATCGTACCTAAAAAGTCATCACCGTACCTAAGGTCATCT  51755

bosTau6_CR_re   5101 AAGTTTTCTCTTATTTTGTCTCCTGGGAGTTTCAAAGTTTTCCATTTTAT   5150
                     ||||||||||||||||||||||||||||||||||||||||||||||||||
CH240-104M22   51756 AAGTTTTCTCTTATTTTGTCTCCTGGGAGTTTCAAAGTTTTCCATTTTAT  51805

bosTau6_CR_re   5151 GTTTAGGTCTACAATCCATTCGGAGTTGGTTTTTGTGAAGGGTGTAAGGT   5200
                     ||||||||||||||||||||||||||||||||||||||||||||||||||
CH240-104M22   51806 GTTTAGGTCTACAATCCATTCGGAGTTGGTTTTTGTGAAGGGTGTAAGGT  51855

bosTau6_CR_re   5201 CTGCGCCTCACGCAGACCTTAGATTCAGTTTTTTGTGGATGTCCGATTGG   5250
                     ||||||||||||||||||||||||||||||||||||||||||||||||||
CH240-104M22   51856 CTGCGCCTCACGCAGACCTTAGATTCAGTTTTTTGTGGATGTCCGATTGG  51905

bosTau6_CR_re   5251 TCCAGCGCCGTTTGTTGACAAGATCATCTTTGCTCCATCCTGTTGCCTCT   5300
                     ||||||||||||||||||||||||||||||||||||||||||||||||||
CH240-104M22   51906 TCCAGCGCCGTTTGTTGACAAGATCATCTTTGCTCCATCCTGTTGCCTCT  51955

bosTau6_CR_re   5301 ACCCCTTGATTGAAGATCAATGACAATATTTGTGGAGCCTATCTGTGTTC   5350
                     ||||||||||||||||||||||||||||||||||||||||||||||||||
CH240-104M22   51956 ACCCCTTGATTGAAGATCAATGACAATATTTGTGGAGCCTATCTGTGTTC  52005

bosTau6_CR_re   5351 TGTATTCCATTCCATTGATCTATTTGTCTTTTCGTTCACCAATTAGACAC   5400
                     ||||||||||||||||||||||||||||||||||||||||||||||||||
CH240-104M22   52006 TGTATTCCATTCCATTGATCTATTTGTCTTTTCGTTCACCAATTAGACAC  52055

bosTau6_CR_re   5401 TGTCTTGATTCGTAGGACTTACGATTTCATGGTTCTGAAAATCTTCCATT   5450
                     ||||||||||||||||||||||||||||||||||||||||||||||||||
CH240-104M22   52056 TGTCTTGATTCGTAGGACTTACGATTTCATGGTTCTGAAAATCTTCCATT  52105

bosTau6_CR_re   5451 AAGTCTTGAAATTTAGTAATGTTAGCCCTCTGACATAGTCCTTCTTCTGT   5500
                     ||||||||||||||||||||||||||.|||||||.|||||||||||||||
CH240-104M22   52106 AAGTCTTGAAATTTAGTAATGTTAGCGCTCTGACTTAGTCCTTCTTCTGT  52155

bosTau6_CR_re   5501 ATTGTACTGACTACTCTGGATTTTTTGTCTCTATGTATAAGAGGAACTAA   5550
                     |||||||||||||.|||||.||||||||||||||||||||||||||||||
CH240-104M22   52156 ATTGTACTGACTATTCTGGGTTTTTTGTCTCTATGTATAAGAGGAACTAA  52205

bosTau6_CR_re   5551 AAAGCCTCTTGATGAAAGTGAAAGAGAAGAGTGAAAAAGTTGGCTTAAAG   5600
                     ||||||||||||||||||||||||||||||||||||||||||||||||||
CH240-104M22   52206 AAAGCCTCTTGATGAAAGTGAAAGAGAAGAGTGAAAAAGTTGGCTTAAAG  52255

bosTau6_CR_re   5601 CTCAACATTCAGAAAACAAATATCATGGCATCTGGTCCCATCACTTCATG   5650
                     ||||||||||||||||||||||||||||||||||||||||||||||||||
CH240-104M22   52256 CTCAACATTCAGAAAACAAATATCATGGCATCTGGTCCCATCACTTCATG  52305

bosTau6_CR_re   5651 GGAAATAGATGGGGAAACAGTGGAAACAGTGAAAGACTTTATTTTCTTGG   5700
                     ||||||||||||||||||||||||||||||||||||||||||||||||||
CH240-104M22   52306 GGAAATAGATGGGGAAACAGTGGAAACAGTGAAAGACTTTATTTTCTTGG  52355

bosTau6_CR_re   5701 GCTCCAAAATCACTGCAGATGGTGACTGCAGCCATGAAATTAAAAGACGC   5750
                     ||||||||||||||||||||||||||||||||||||||||||||||||||
```

```
CH240-104M22   52356 GCTCCAAAATCACTGCAGATGGTGACTGCAGCCATGAAATTAAAAGACGC  52405

bosTau6_CR_re   5751 TTACTCCTTGGAAGGAAAGTTATGACCAACCTAGATAGCATATTGAAAAG   5800
                     ||||||||||||||||||||||||||||||||||||||||||||||||||
CH240-104M22   52406 TTACTCCTTGGAAGGAAAGTTATGACCAACCTAGATAGCATATTGAAAAG  52455

bosTau6_CR_re   5801 CAGAGACATTACTTTGCCAACAAAGGTCCATCTAGTCAAAGCTATGGTTT   5850
                     ||||||||||||||||||||||||||||||||||||||||||||||||||
CH240-104M22   52456 CAGAGACATTACTTTGCCAACAAAGGTCCATCTAGTCAAAGCTATGGTTT  52505

bosTau6_CR_re   5851 TTCCTGTGGTCATATATGGATGTGAGAGTTGGACTGTGAAGAAGGCTGAG   5900
                     ||||||||||||||||||||||||||||||||||||||||||||||||||
CH240-104M22   52506 TTCCTGTGGTCATATATGGATGTGAGAGTTGGACTGTGAAGAAGGCTGAG  52555

bosTau6_CR_re   5901 CACCGAAGAATTGATGCTTTTGAACTGTGGTGTTGGAGAAGACTCTTGAG   5950
                     ||||||||||||||||||||||||||||||||||||||||||||||||||
CH240-104M22   52556 CACCGAAGAATTGATGCTTTTGAACTGTGGTGTTGGAGAAGACTCTTGAG  52605

bosTau6_CR_re   5951 AGTCCCTTGGACTGCAAGGAGATCCAACCAGTCCATTCTGAAGGAGATCA   6000
                     ||||||||||||||||||||||||||||||||||||||||||||||||||
CH240-104M22   52606 AGTCCCTTGGACTGCAAGGAGATCCAACCAGTCCATTCTGAAGGAGATCA  52655

bosTau6_CR_re   6001 G   6001
                     |
CH240-104M22   52656 G  52656
```
