# Supplementary figures and images for: Remapping of the belted phenotype in cattle on BTA3 identifies a multiplication event as the candidate causal mutation
Source: Genet Sel Evol. 2018 Jul 6;50:36. doi: 10.1186/s12711-018-0407-9 (PMC6035435; doi:10.1186/s12711-018-0407-9)

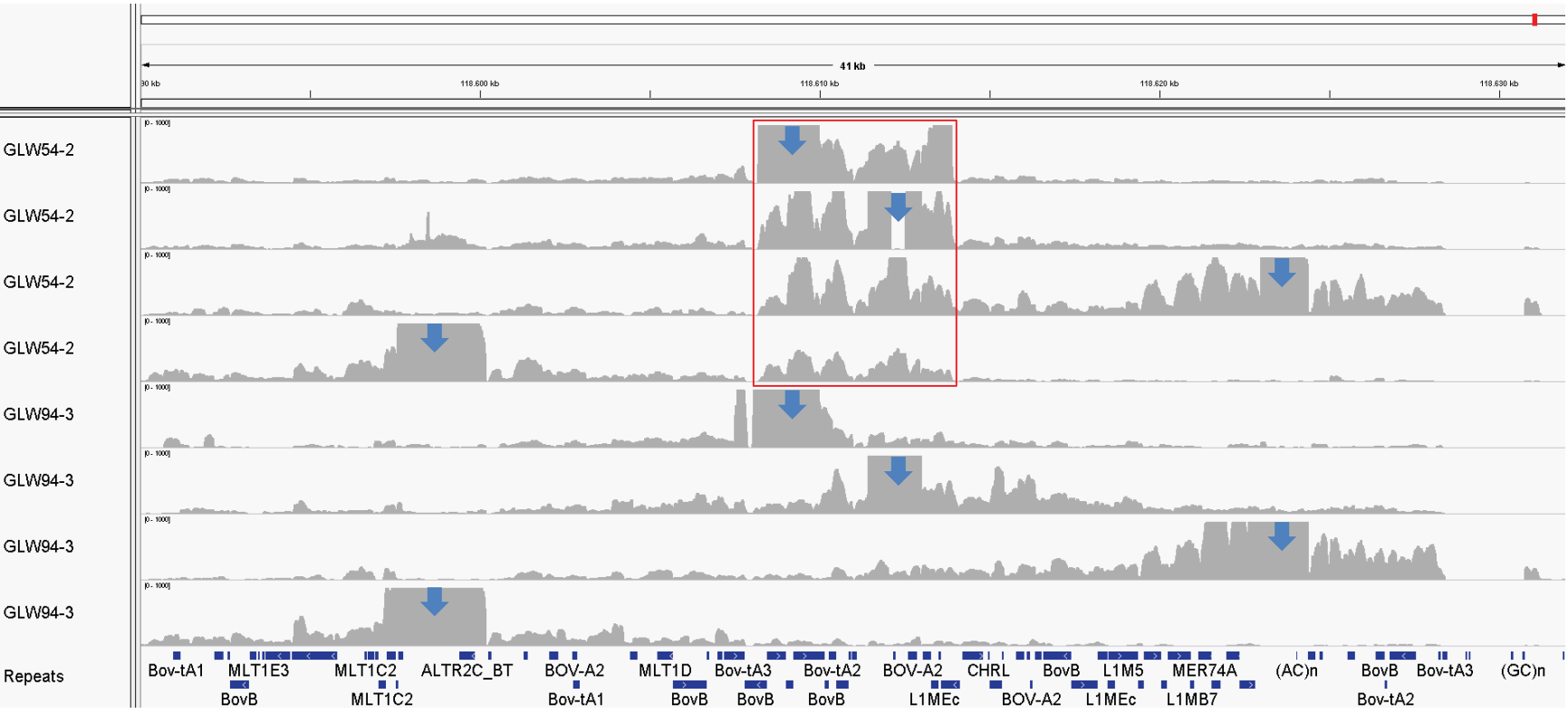

Supplement: Supplementary file 3 — Additional file 3. TLA results. The genomic region chr3:118,590,000–118,632,000 (bosTau8) is displayed. The arrows indicate the position of the primer sets used for TLA. For the belted animal (GLW54-2), an increased copy number (3 to 4.5 times) was detected in the region indicated by the red rectangle. The y-axis is limited to max. 1000X. [file 12711_2018_407_MOESM3_ESM.pdf]

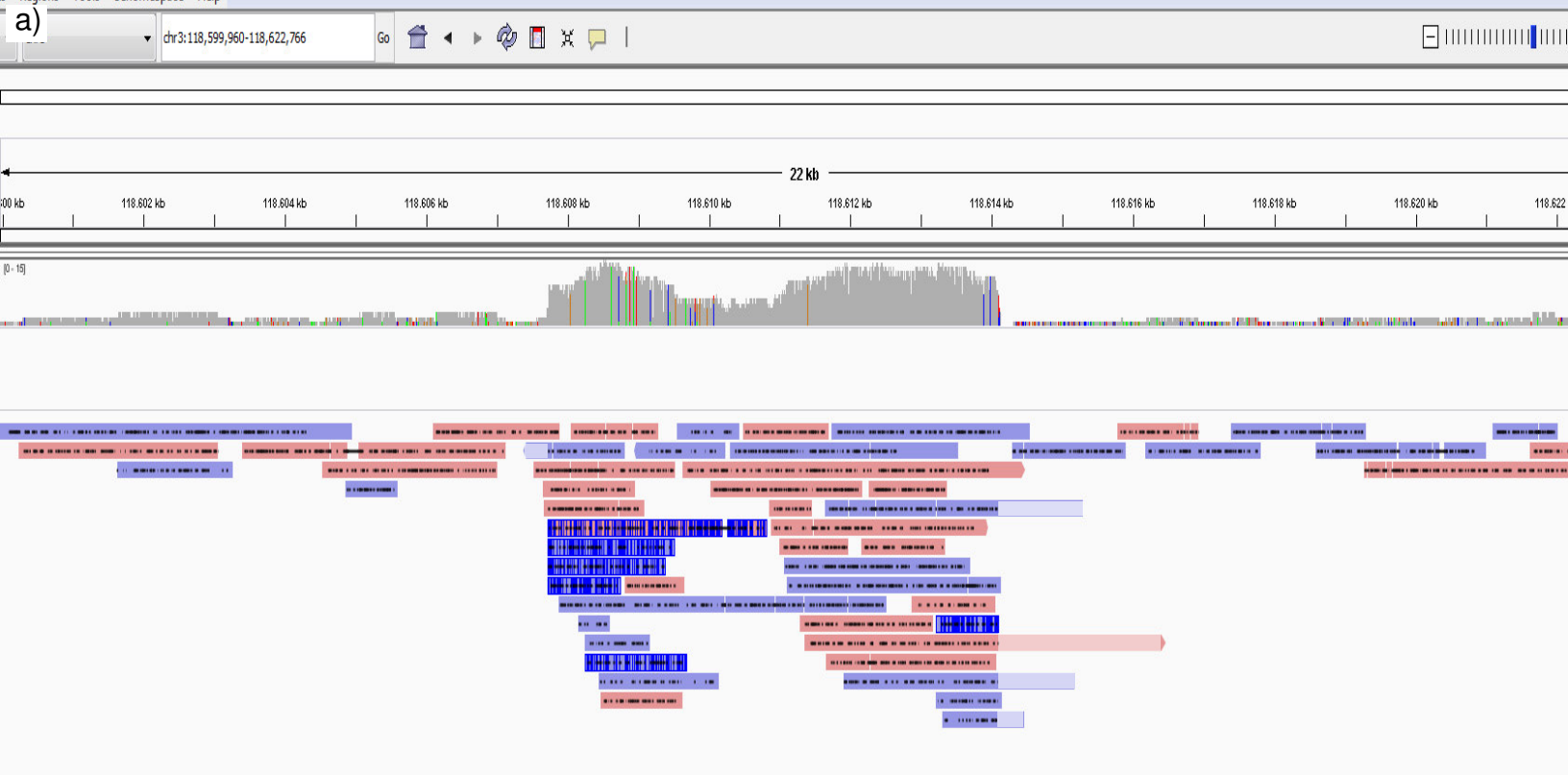

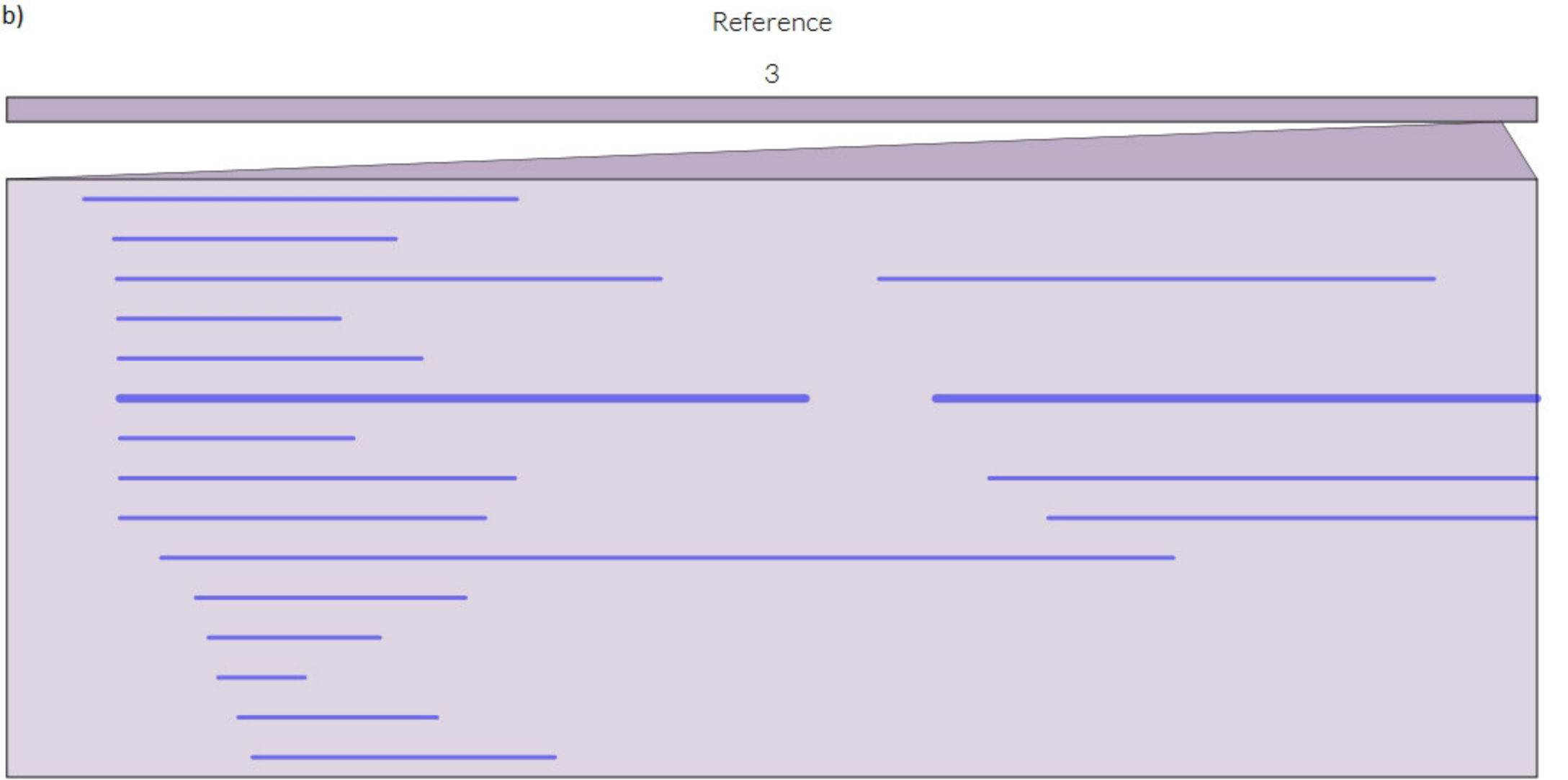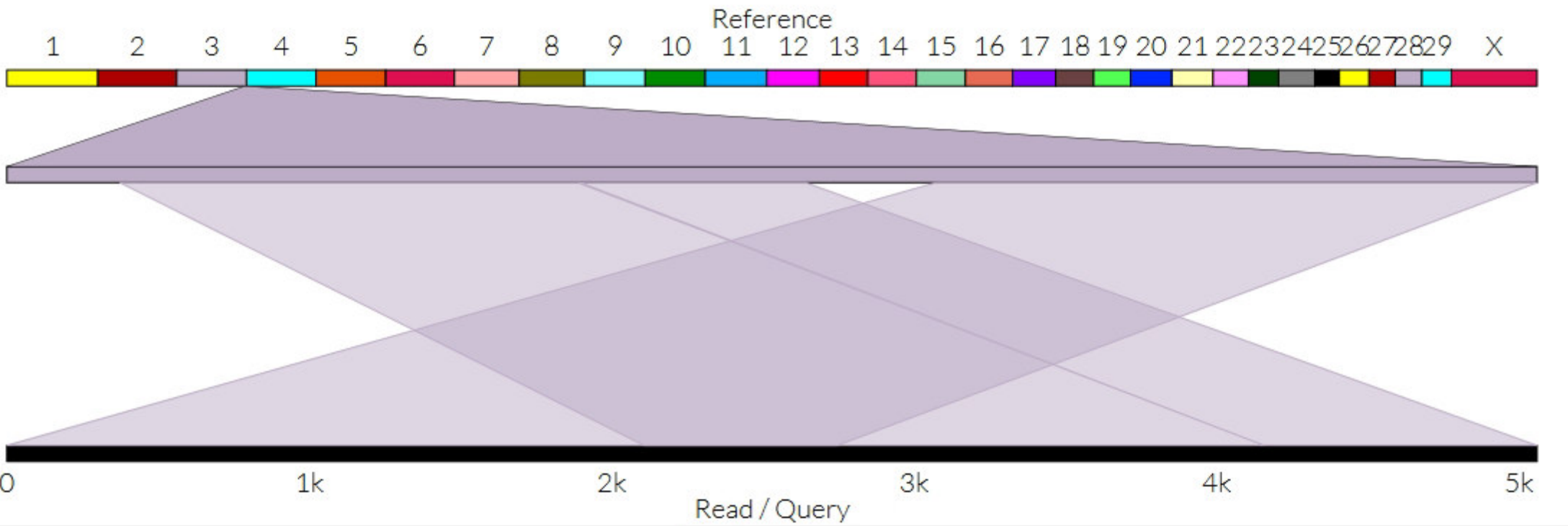

c)

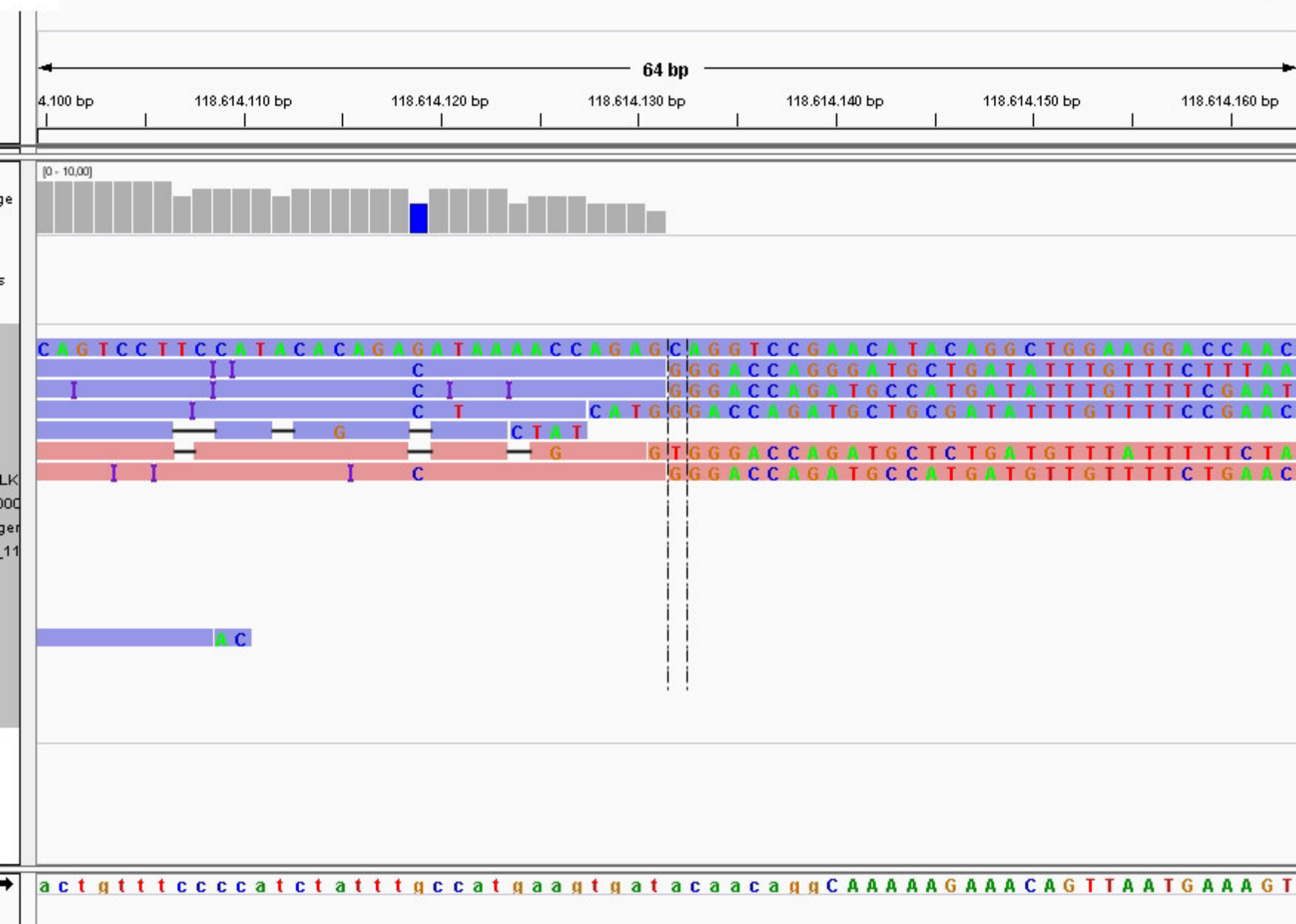

Supplement: Supplementary file 5 — Additional file 5. Nanopore sequencing results. (a) Nanopore reads mapped (minimap2) to the belted candidate region are shown. The shaded parts of the reads highlight unmapped portions of the read that were mapped as a secondary alignment in a separate read. These secondary alignments are highlighted by blue borders. (b) Split-alignment of breakpoint-spanning read visualized by Ribbon [52]. The highlighted read (bold blue line) is shown as a zoom in the lower panel, showing that the beginning of the read is found at the end of the repeated region and the end is found at the beginning, thus illustrating the concatenation of the repeat units found in belted cattle. (c) Exact breakpoints were identified by inspection of the partially mapped reads. The right breakpoint at 118,614,132 bp shows that the unmapped portion of the split-aligned reads starts with a sequence that is located at 118,608,362 bp, which thus defines the left breakpoint. [file 12711_2018_407_MOESM5_ESM.pdf]

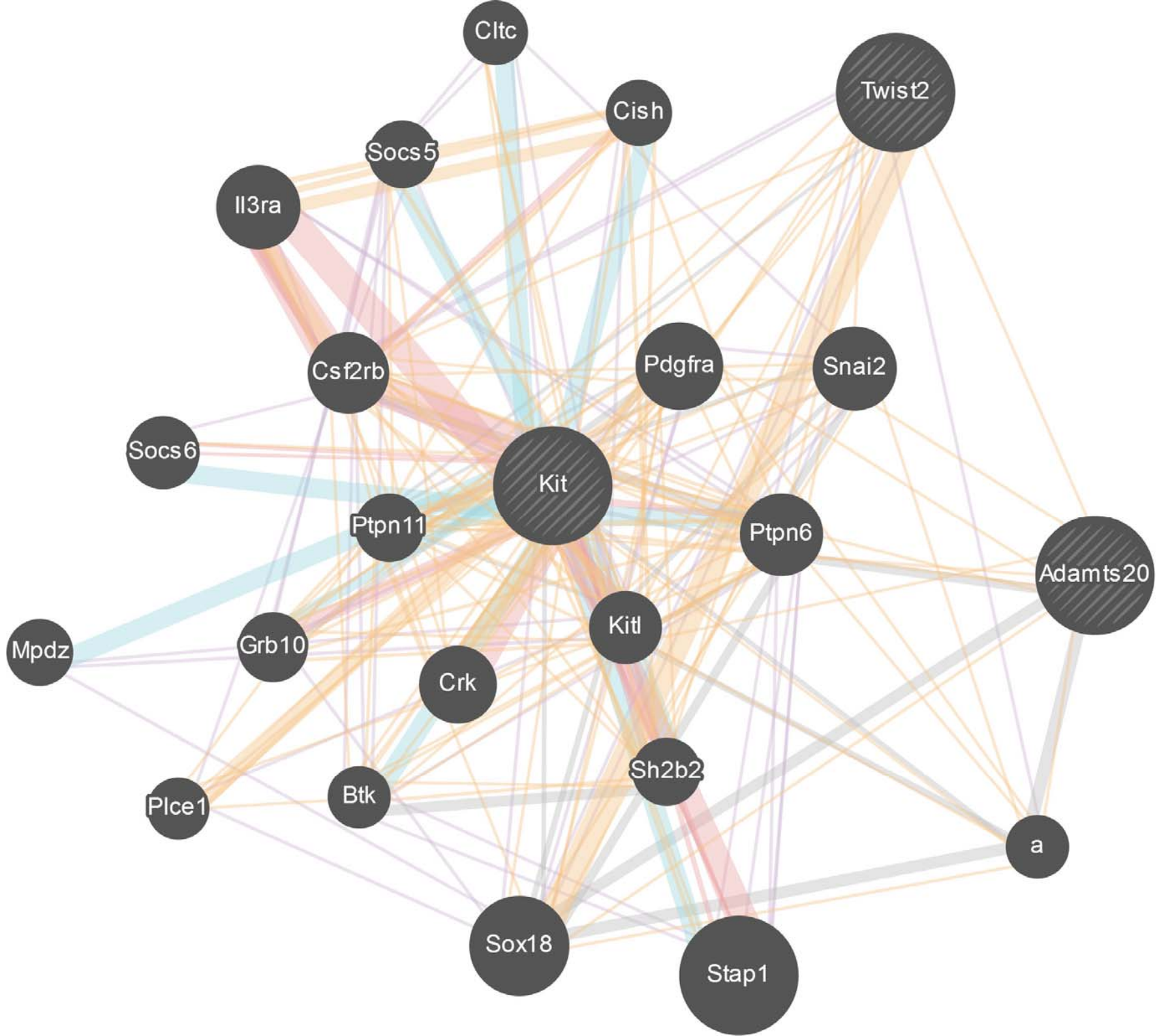

Supplement: Supplementary file 7 — Additional file 7. Gene interaction network. This figure illustrates the interactions between KIT (causal for the belt in pigs), ADAMTS20 (causal for the belt in mice) and TWIST2 (most likely causal for the belt in cattle) in mice. Interaction line colors are as follows: orange: predicted functional relationship, red: physical interactions, purple: co-expression; grey: phenotype (based on mouse genome informatics) and blue: participation in the same reaction within a pathway. [file 12711_2018_407_MOESM7_ESM.pdf]
