# Supplementary material for: Remapping of the belted phenotype in cattle on BTA3 identifies a multiplication event as the candidate causal mutation
Source: Genet Sel Evol. 2018 Jul 6;50:36. doi: 10.1186/s12711-018-0407-9 (PMC6035435; doi:10.1186/s12711-018-0407-9)
Supplement: Supplementary file 4 — Additional file 4. Alignment of the repetitive elements at the beginning and end of the 6-kb candidate segment. Pairwise alignment of the reference sequences (BosTaurus8) of LINE BovB at the beginning of the 6-kb candidate segment and LINE BovB at the end of the segment. This file shows the huge level of similarity between the start and end of the candidate segment as is also obvious in Fig. 2d. [file 12711_2018_407_MOESM4_ESM.pdf]

```
#=======================================
#
# Aligned_sequences: 2
# 1: BovB_start
# 2: BovB_end
# Matrix: EDNAFULL
# Gap_penalty: 10.0
# Extend_penalty: 0.5
#
# Length: 668
# Identity:     541/668 (81.0%)
# Similarity:   541/668 (81.0%)
# Gaps:         104/668 (15.6%)
# Score: 2577.5
#
#
#=======================================

BovB_start         1 AAGAGGAACTAAAAAGCCTCTTGATGAAAGTGAAAGAGAAGAGTGAAAAA
50

BovB_end           1 --------------------------------------------------
0

BovB_start        51 GTTGGCTTAAAGCTCAACATTCAGAAAACAAATATCATGGCATCTGGTCC
100
                                                             |.|.|.||
BovB_end           1 ----------------------------------------CCTGTTGT--
8

BovB_start       101 CATCACTTCATGGGAAATAGATGGGGAAACAGTGGAAACAGTGAAAGACT
150
                      ||||||||||||.||||||||||         ||||||||||..|||||
BovB_end           9 -ATCACTTCATGGCAAATAGATGG---------GGAAACAGTGTCAGACT
48

BovB_start       151 TTATTTTCTTGGGCTCCAAAATCACTGCAGATGGTGACTGCAGCCATGAA
200
                     |||||||.||||||||||||||||||||||||||||||||||||||||||
BovB_end          49 TTATTTTTTTGGGCTCCAAAATCACTGCAGATGGTGACTGCAGCCATGAA
98

BovB_start       201 ATTAAAAGACGCTTACTCCTTGGAAGGAAAGTTATGACCAACCTAGATAG
250
                     |||||||||.||||||||||||||||||||||||||||||||||||||||
BovB_end          99 ATTAAAAGATGCTTACTCCTTGGAAGGAAAGTTATGACCAACCTAGATAG
148

BovB_start       251 CATATTGAAAAGCAGAGACATTACTTTGCCAACAAAGGTCCATCTAGTCA
300
                     ||||||.|||||||||||.|||||||||||||||||||||||||||||||
BovB_end         149 CATATTCAAAAGCAGAGATATTACTTTGCCAACAAAGGTCCATCTAGTCA
198

BovB_start       301 AAGCTATGGTTTTTCCTGTGGTCATATATGGATGTGAGAGTTGGACTGTG
350
                     |.||  ||||||||||.||||||||.||||||||||||||||||||||||
BovB_end         199 AGGC--TGGTTTTTCCAGTGGTCATGTATGGATGTGAGAGTTGGACTGTG
246

BovB_start       351 AAGAAGGCTGAGCACCGAAGAATTGATGCTTTTGAACTGTGGTGTTGGAG
400
                     |||||..|||||||||||||||||.|||||||||||||||||||||||||
BovB_end         247 AAGAAAACTGAGCACCGAAGAATTAATGCTTTTGAACTGTGGTGTTGGAG
296

BovB_start       401 AAGACTCTTGAGAGTCCCTTGGACTGCAAGGAGATCCAACCAGTCCATTC
```

```
450
                        |||||||||||||.|||||||||||||||||||||.||||||||||||||
BovB_end          297 AAGACTCTTGAGACTCCCTTGGACTGCAAGGAGATGCAACCAGTCCATTC
346

BovB_start        451 TGAAGGAGATCAGCCCTGGGATTTCTTTGGAAGGGATAATGCTAAAGCTG
500
                        |.||||||||||||||||||||||||||||||||.|||||||||||||||
BovB_end          347 TAAAGGAGATCAGCCCTGGGATTTCTTTGGAAGGAATAATGCTAAAGCTG
396

BovB_start        501 AAACTCCAGTACTTTGGCCACCTGATGTGAAGAGTTGACTCATTGGAAAA
550
                        ||||||||||||||||||||||||||||||||||||||||||||||||||
BovB_end          397 AAACTCCAGTACTTTGGCCACCTGATGTGAAGAGTTGACTCATTGGAAAA
446

BovB_start        551 GACTCTGATGCTGGGAGGGATTGGGGGCAGGAGGAGAAGGGGACGACAGA
600
                        ||||||||||||||||||||||||||||||||||||||||||||||||||
BovB_end          447 GACTCTGATGCTGGGAGGGATTGGGGGCAGGAGGAGAAGGGGACGACAGA
496

BovB_start        601 GGATGAGATGGCTGGATGGCATCACTGACTCGATGGACGTGAGTCTGAGT
650
                        |||||||||||||||||||||||||.||||||||||||||||||||||||
BovB_end          497 GGATGAGATGGCTGGATGGCATCACCGACTCGATGGACGTGAGTCTGAGT
546

BovB_start        651 GAACTCCAGGAGTTGCTG    668
                        |||||||.|||||||.||
BovB_end          547 GAACTCCGGGAGTTGGTG    564


#---------------------------------------
#---------------------------------------
```
