# Supplementary material for: Remapping of the belted phenotype in cattle on BTA3 identifies a multiplication event as the candidate causal mutation
Source: Genet Sel Evol. 2018 Jul 6;50:36. doi: 10.1186/s12711-018-0407-9 (PMC6035435; doi:10.1186/s12711-018-0407-9)
Supplement: Supplementary file 6 — Additional file 6. Alignment of the repetitive elements at the beginning of the 6-kb candidate segment according to bosTaurus6 and bosTaurus8. The pairwise alignment of the reference sequence of the SINE element ART2A (bosTau6) and the LINE element BovB (bosTau8) at the beginning of the 6-kb candidate segment shows that ART2A is part of BovB. [file 12711_2018_407_MOESM6_ESM.pdf]

```
#=======================================
#
# Aligned_sequences: 2
# 1: bosTau6_ART2A
# 2: bosTau8_BovB
# Matrix: EDNAFULL
# Gap_penalty: 10.0
# Extend_penalty: 0.5
#
# Length: 752
# Identity:     453/752 (60.2%)
# Similarity:   453/752 (60.2%)
# Gaps:         299/752 (39.8%)
# Score: 2265.0
#
#
#=======================================

bosTau6_ART2A      1 --------------------------------------------------
0

bosTau8_BovB       1 aagaggaactaaaaagcctcttgatgaaagtgaaagagaagagtgaaaaa
50

bosTau6_ART2A      1 --------------------------------------------------
0

bosTau8_BovB      51 gttggcttaaagctcaacattcagaaaacaaatatcatggcatctggtcc
100

bosTau6_ART2A      1 --------------------------------------------------
0

bosTau8_BovB     101 catcacttcatgggaaatagatggggaaacagtggaaacagtgaaagact
150

bosTau6_ART2A      1 --------------------------------------------------
0

bosTau8_BovB     151 ttattttcttgggctccaaaatcactgcagatggtgactgcagccatgaa
200

bosTau6_ART2A      1 ---------------ctccttggaaggaaagttatgaccaacctagatag
35
                                    |||||||||||||||||||||||||||||||||||
bosTau8_BovB     201 attaaaagacgcttactccttggaaggaaagttatgaccaacctagatag
250

bosTau6_ART2A     36 catattgaaaagcagagacattactttgccaacaaaggtccatctagtca
85
                     ||||||||||||||||||||||||||||||||||||||||||||||||||
bosTau8_BovB     251 catattgaaaagcagagacattactttgccaacaaaggtccatctagtca
300

bosTau6_ART2A     86 aagctatggtttttcctgtggtcatatatggatgtgagagttggactgtg
135
                     ||||||||||||||||||||||||||||||||||||||||||||||||||
bosTau8_BovB     301 aagctatggtttttcctgtggtcatatatggatgtgagagttggactgtg
350

bosTau6_ART2A    136 aagaaggctgagcaccgaagaattgatgcttttgaactgtggtgttggag
185
                     ||||||||||||||||||||||||||||||||||||||||||||||||||
bosTau8_BovB     351 aagaaggctgagcaccgaagaattgatgcttttgaactgtggtgttggag
400

bosTau6_ART2A    186 aagactcttgagagtcccttggactgcaaggagatccaaccagtccattc
```

```
235
                             ||||||||||||||||||||||||||||||||||||||||||||||||||
bosTau8_BovB      401 aagactcttgagagtcccttggactgcaaggagatccaaccagtccattc
450

bosTau6_ART2A     236 tgaaggagatcagccctgggatttctttggaagggataatgctaaagctg
285
                             ||||||||||||||||||||||||||||||||||||||||||||||||||
bosTau8_BovB      451 tgaaggagatcagccctgggatttctttggaagggataatgctaaagctg
500

bosTau6_ART2A     286 aaactccagtactttggccacctgatgtgaagagttgactcattggaaaa
335
                             ||||||||||||||||||||||||||||||||||||||||||||||||||
bosTau8_BovB      501 aaactccagtactttggccacctgatgtgaagagttgactcattggaaaa
550

bosTau6_ART2A     336 gactctgatgctgggagggattgggggcaggaggagaaggggacgacaga
385
                             ||||||||||||||||||||||||||||||||||||||||||||||||||
bosTau8_BovB      551 gactctgatgctgggagggattgggggcaggaggagaaggggacgacaga
600

bosTau6_ART2A     386 ggatgagatggctggatggcatcactgactcgatggacgtgagtctgagt
435
                             ||||||||||||||||||||||||||||||||||||||||||||||||||
bosTau8_BovB      601 ggatgagatggctggatggcatcactgactcgatggacgtgagtctgagt
650

bosTau6_ART2A     436 gaactccaggagttgctgatggacagggaggcctggcgtgctgtgattca
485
                             ||||||||||||||||||
bosTau8_BovB      651 gaactccaggagttgctg--------------------------------
668

bosTau6_ART2A     486 tggggtcacagagtcggacacaactgagcgactgaactgacggactgact
535

bosTau8_BovB      669 --------------------------------------------------
668

bosTau6_ART2A     536 ga     537

bosTau8_BovB      669 --     668


#---------------------------------------
#---------------------------------------
```
